# Intersection of Precision Nutrition and Bladder Cancer: A Narrative State-of-the-Art Review of Potential Applications and Challenges

**DOI:** 10.3390/jcm15031247

**Published:** 2026-02-04

**Authors:** Tevfik Koçak, Yağmur Demirel Özbek, Mahmut Bodur, Süleyman Yeşil, Duygu Ağagündüz

**Affiliations:** 1 Department of Nutrition and Dietetics, Gümüşhane University, Merkez, Gümüşhane 29100, Turkey; tkocak@gumushane.edu.tr; 2 Department of Nutrition and Dietetics, Faculty of Health Sciences, Recep Tayyip Erdoğan University, Rize 53100, Turkey; 3 Department of Nutrition and Dietetics, Faculty of Health Sciences, Ankara University, Ankara 06000, Turkey; mahmutbodur@ankara.edu.tr; 4 Department of Urology, School of Medicine, Gazi University, Ankara 06490, Turkey; syesil2003@yahoo.com; 5 Department of Nutrition and Dietetics, Gazi University, Emek, Ankara 06490, Turkey

**Keywords:** bladder cancer, precision nutrition, nutrigenomics, precision diet, bladder cancer prevention, metabolomics, digital health

## Abstract

Bladder cancer (BC) is a biologically heterogeneous tumor affected by genetic, metabolic, environmental, and lifestyle factors. Recent research indicates that nutrition can change the way urothelial cancer forms by affecting inflammation, oxidative stress, cellular energy, and the epigenome. It can also change the risk of BC and how well treatment works. Simultaneous progress in precision nutrition (PN) and nutriomic profiling—encompassing nutrigenomics, nutrigenetics, nutriepigenetics, metabolomics, and microbiome science—presents novel options to tailor dietary regimens beyond universal guidelines. In this review, we consolidate existing knowledge regarding the nutritional factors influencing BC, outline pertinent principles of PN for BC prevention and survival, and explore how urine proteomics and molecular subtyping facilitate the integration of PN into precision oncology. Our review examines the methodological, bioinformatic, biomarker, and clinical translation challenges that impede the implementation of PN in BC management; these challenges include the need for validated nutritional biomarkers with mechanistic endpoints, interoperable data platforms, and rigorously designed clinical trials. Finally, we emphasize future prospects for PN-guided medical nutrition therapy and dietary models during and after systemic treatment recovery. We propose research priorities that will facilitate the integration of PN-informed individualized dietary plans with medical and surgical approaches in BC treatment, aiming to decrease the costs associated with expensive or excessively aggressive treatment methods, thereby supporting long-term survival care. This review seeks to establish a conceptual framework for the integration of PN into BC management by delineating the opportunities and challenges, hence promoting hypothesis-driven research in a promising yet underexplored domain.

## 1. Introduction

Bladder cancer (BC), which is predominantly a urothelial carcinoma, is a major cause of cancer-related morbidity and mortality worldwide. It is characterized by significant biological, histological and clinical heterogeneity, which complicates prevention, diagnosis and treatment [1,2,3]. In addition to its general prevalence, the disease includes several different forms that differ in terms of their structural features, mutational load, immune environment, and clinical progression. These differences necessitate a more nuanced approach than traditional uniform care pathways [1,4]. Bladder carcinogenesis is multifactorial in terms of its etiology: environmental carcinogens and lifestyle factors can interact with aging biology and host comorbidity, thereby initiating and promoting tumor evolution. The high mutational burden typical of urothelial tumors also underscores their complex molecular pathogenesis [1,4].

Pathologically, the urothelial spectrum ranges from non-muscle-invasive bladder cancer (NMIBC) to muscle-invasive bladder cancer (MIBC). Molecular taxonomies (e.g., luminal–basal lineages and immune “hot/cold” phenotypes) have refined prognostic stratification and therapeutic decision-making [4]. Multimodal "omics" profiling has facilitated this reclassification and begun to bridge the gap between histopathology and function. This includes genomic alterations (e.g., FGFR3) and immune signatures that can predict the effectiveness of systemic therapies [2,4]. At the proteome level, studies highlight panels and extracellular vesicles that can be used to diagnose and predict tumor presence and severity. These provide easy-to-implement tissue genomic markers [5,6,7]. Urinary proteomic panels, in particular, have achieved promising sensitivity and specificity in multicenter validations, with exosome-derived proteins such as Alpha-1 Antitrypsin (A1AT), histone H2B (H2B) variants and periostin emerging as candidates for risk stratification and surveillance in NMIBC and MIBC [5,7].

Precision oncology and medicine in BC is based on these biological insights, with the main goal in this field being to match molecular features with targeted and immunotherapeutic strategies. Despite these advances, current nutritional recommendations in BC largely rely on population-based, generalized dietary guidelines that do not sufficiently account for inter-individual variability in genetic background, metabolic phenotype, environmental exposures, or lifestyle-related risk factors. Such “one-size-fits-all” approaches may therefore fail to adequately address the complex and multifactorial etiology of bladder carcinogenesis, where diet interacts dynamically with host metabolism, immune function, and molecular tumor characteristics. This limitation underscores the need for individualized, biology-driven nutritional strategies capable of adapting dietary interventions to patient-specific molecular and metabolic profiles [5].

However, despite the abundance of potential therapeutic targets, the clinical translation of biomarker-guided therapies has been inconsistent, highlighting the need for rigorous validation, robust trial designs and comprehensive longitudinal data [1,2,4]. In the context of individualized cancer care, precision nutrition (PN) emerges as a complementary approach. Integrating nutriomic profiles, which include nutrigenomics, nutrigenetics, nutriepigenetics, nutrimetabolomics and nutrimetagenomics, with clinical, psychosocial and environmental factors, PN seeks to personalize dietary recommendations based on individual biology, moving beyond the limitations of “one-size-fits-all” advice [8]. A recent international position statement further supports this view, underlining that advances in bioinformatics and machine learning now make it possible to translate complex nutrigenomic profiles into practical, disease-specific nutrition strategies [8]. There is a link between the development of cancer and PN. The diet affects redox homeostasis, inflammation, cellular energetics and the epigenome, all of which are important factors in cancer. These diet–host interactions can be measured using multi-omics approaches [9].

Advances in nutrigenomic research and microbiome science over the last two decades have led to the development of PN frameworks that now support testable hypotheses. These hypotheses identify who benefits from specific dietary modifications and how to maintain those changes. They also include real-time monitoring and AI-assisted adaptation [9]. In BC specifically, the maturity of urinary proteomics provides a practical pathway for PN, as urinary peptide and protein signatures are already informative for diagnosis, progression, and recurrence. Urinary proteomic markers also function as minimally invasive indicators to evaluate nutritional exposures and responses that influence urothelial tumor biology [5,6,7,10]. The future direction of research in this field is expected to involve the integration of molecular BC subtypes and immune phenotypes with nutriomic profiles and microbiome states. This integration is expected to improve risk classification, provide support for resilience during treatment such as intravesical therapy or precystectomy care, and facilitate the development of long-term survivorship plans. However, integrating PN into BC care has its challenges. These include the heterogeneity of urothelial tumors and patient populations; the need for validated, disease-specific nutritional biomarkers that map to mechanistic endpoints; methodological rigor in study design with regard to confounding factors, adherence and endpoints; and interoperable platforms that integrate dietary, clinical and multi-omics data at the individual level [2,8,9]. This article is a narrative, state-of-the-art review that synthesizes mechanistic, translational, and emerging clinical evidence at the intersection of precision nutrition and bladder cancer. This narrative review seeks to elucidate the nascent intersection of precision medicine and oncology, with precision nutrition in BC, and to assess its translational viability. The study aims to elucidate how precision dietary strategies may enhance modern diagnostic and therapeutic approaches by integrating advancements in molecular BC subtyping, urine proteomics, and nutriomic profiling. The study delineates essential research deficiencies, including biomarker validation, mechanistic endpoints, and data integration requirements, to facilitate the advancement of disease-specific dietary therapies and to guide forthcoming hypothesis-driven clinical investigations.

## 2. Nutrition and Bladder Cancer

Nutrition is seen as a modifiable environmental variable in cancer progression. In recent years, its association with BC has been the focus of increased research [11]. The direct interaction of the bladder mucosa with compounds from dietary macro- and micronutrients makes this association especially significant in comparison to that with other cancer types. Numerous epidemiological studies have demonstrated that dietary habits can influence BC risk both favorably and adversely [12,13]. Diets rich in fruits and vegetables, which are characterized by antioxidant and anti-inflammatory components, have been linked to a lower BC risk [14]. In contrast, dietary patterns characterized by a high glycemic index (GI), refined carbohydrates, saturated fats, and processed foods have been associated with carcinogenesis-related outcomes [13]. In cases of BC, dietary choices before and after diagnosis may influence tumor progression due to the direct interaction of excreted metabolites with the bladder’s urothelium.

### 2.1. Macronutrients

#### 2.1.1. Carbohydrates

Carbohydrates serve as the principal energy source in the diet, and their quality can influence health through metabolic processes [15]. The association between carbohydrates and BC is frequently evaluated by using measurements of GI and glycemic load (GL). Foods with a high GI are rapidly digested and absorbed, resulting in rapid increases in blood glucose levels [16]. This process enhances insulin secretion from the pancreas and can result in hyperinsulinemia over time. Hyperinsulinemia enhances the bioavailability of insulin-like growth factor 1 (IGF-1), thereby promoting cell proliferation and inhibiting apoptosis. This results in carcinogenesis [16,17]. Ingesting high-GI foods may induce oxidative stress and chronic inflammation, resulting in Deoxyribonucleic Acid (DNA) damage and genetic alterations [18]. BC cells, like many other cancer cells, show the Warburg effect in their energy metabolism. This means that they prefer glycolysis, even when oxygen is available. This metabolic shift not only generates energy but also facilitates the biosynthesis of nucleotides, lipids, amino acids, and nucleotide sugars, all of which are critical for tumor growth and expansion [19]. The hexosamine biosynthesis pathway, a part of carbohydrate metabolism, uses glucose to make UDP-N-acetylglucosamine. This compound triggers an increase in protein modifications known as O-GlcNAcylation. O-GlcNAcylation promotes cell proliferation, inflammation, and metastatic capabilities through the modulation of several oncogenic factors, such as nuclear factor-kappa B (NF-κB), HIF-1α, MYC, and β-catenin [20]. This process alters glycan structures on the cell surface by affecting the activity of glycosyltransferase enzymes within tumor cells. Consequently, this enhances cellular adhesion, motility, and immune evasion mechanisms [21].

Glycobiological alterations are particularly pronounced in BC. Significant differences are observed in the N-glycan and O-glycan structures present on the surfaces of tumor cells. The enhanced β1-6 branching in N-glycans facilitates the clustering of cell surface receptors, thereby intensifying intracellular signaling [22]. Furthermore, this branching contributes to the spread and metastasis of cancerous cells. In the case of O-glycans, premature termination results in the accumulation of short-chain Tn, sTn, and T antigens. These antigens are generally associated with tumor progression, poor prognosis, and immune system evasion [20].

Epidemiological studies have not consistently demonstrated a direct association between total carbohydrate intake and BC risk. However, dietary patterns characterized by higher GI or GL have been suggested as potential risk-related exposures [16,17,18]. These findings indicate that carbohydrate quality and source, rather than absolute carbohydrate intake, may better reflect carbohydrate-related dietary patterns relevant to BC risk.

#### 2.1.2. Dietary Fiber

Dietary fiber is a polysaccharide that is indigestible in the gastrointestinal system but is capable of fermentation in the intestines. Legumes, whole grains, vegetables, and fruits constitute the principal sources. It is crucial for intestinal health and specifically improves digestive function. Besides its significant and recognized function, fiber has been documented to confer protective benefits against some cancer types [23,24].

The association between fiber and BC can be explained through various biological mechanisms. Mechanistically, foods high in fiber decrease the absorption of cancer-causing substances in the gastrointestinal tract [25], thereby indirectly reducing the production of harmful compounds released in urine and interacting with the bladder mucosa [24,26]. Additionally, fiber fermentation produces short-chain fatty acids (SCFAs), including butyrate, which may possess anticancer characteristics and influence cell proliferation. Beyond their direct metabolic effects, SCFAs play a key role in shaping the gut microbiome–immune axis. The production of SCFAs through microbial fermentation has been shown to enhance regulatory T-cell responses, reduce systemic inflammatory signaling, and support immune homeostasis [27], which may indirectly influence the bladder immune microenvironment. These metabolites function as inhibitors of histone deacetylase enzymes, which in turn activates tumor-suppressor genes like p21 and p27. Furthermore, they suppress NF-κB and signal transducer and activator of transcription 3 signaling, leading to a decrease in the production of inflammatory cytokines. SCFAs initiate the Nrf2 pathway, thereby increasing the expression of antioxidant and detoxifying genes, such as HO-1, NQO1, and Glutathione S-transferase Mu 1 (GSTM1). This process ultimately reduces oxidative DNA damage and the subsequent accumulation of mutations [27].

Furthermore, fiber contributes to the enhancement of glycemic regulation. Fiber-rich diets mitigate postprandial glycemic responses and avert sudden changes in insulin and insulin-like growth factor 1 (IGF-1) levels. This pathway may restrict hyperinsulinemia and IGF-1-induced cellular proliferation [16,28]. The elevation of IGF-1, a consequence of hyperinsulinemia, fosters DNA synthesis, cellular proliferation, and angiogenesis through the activation of MAPK and Akt signaling pathways, mediated by the IGF-1 receptor. Dietary fiber intake, which regulates this axis, results in diminished mTORC1 activation and augmented AMPK pathway stimulation; AMPK activation, functioning as an energy sensor, subsequently inhibits cell proliferation, facilitates p53 stabilization, and promotes the elimination of damaged cells via autophagy [16]. Epidemiological evidence indicates that higher dietary fiber intake is associated with a lower risk of BC. Consequently, dietary fiber has been recognized as a significant protective nutrient against BC [26,29].

#### 2.1.3. Fats

Fats are essential nutrients due to their physiological impacts. Research investigating the correlation between BC and dietary fats shows that the specific type of fat ingested is a crucial factor [12]. Saturated fatty acids (SFAs) and trans fatty acids (TFAs) increase inflammatory processes and enhance oxidative stress. This may lead to DNA damage and carcinogenesis [30,31]. Diets characterized by a high SFA content, such as the Western dietary pattern, have therefore been associated with an increased risk of bladder carcinogenesis in observational analyses [12]. SFAs and TFAs may, in some cases, contribute to increased levels of reactive oxygen species (ROS), thereby influencing oxidative stress and related biological functions [12]. Free radicals assault healthy cells, resulting in peroxidation and ultimately causing DNA damage. Consequently, ROS can precipitate tumor initiation and facilitate the advancement of cancer cells [32].

Cholesterol has received increasing attention in recent years because of its role in cancer development. Clinical and experimental research indicates that elevated circulating cholesterol levels are associated with an increased risk of BC [12,33]. Numerous mechanisms have been suggested to elucidate the potential role of cholesterol in cancer progression, including (a) alterations in lipid and apolipoprotein concentrations that may induce cellular inflammation by elevating proinflammatory cytokines, such as tumor necrosis factor-α and interleukin-6 [34], and (b) the distribution of cholesterol balance via the elevation of mitochondrial cholesterol levels, resulting in resistance to apoptotic signals due to the disruption of the cholesterol pathway [12,33].

Monounsaturated fatty acids (MUFAs) and omega-3 polyunsaturated fatty acids (PUFAs) possess antioxidant and anti-inflammatory characteristics. MUFAs regulate membrane fluidity and signaling, mitigate the NF-κB-mediated inflammatory response and oxidative stress induced by SFAs and oxidized fats, and inhibit insulin resistance and proliferative signals through peroxisome proliferator-activated receptor-α/γ activation. Additionally, they downregulate the expression of oncogenic genes such as HER2, FASN, and PEA3 [35].

Omega-3 competes with arachidonic acid at the COX/LOX level, resulting in diminished pro-inflammatory eicosanoid synthesis; mitigates chronic inflammation through soluble mediators; and inhibits NF-κB-derived cytokine and growth factor receptor signaling [12,35]. Monounsaturated fatty acids and omega-3 PUFAs possess antioxidant and anti-inflammatory characteristics, potentially enhancing their preventive effects against cancer. Olive oil, a major source of MUFAs and a central component of the Mediterranean diet, has been inversely associated with BC risk [12,36,37]. In a Belgian case–control study, olive oil consumption was found to be inversely associated with BC risk. In this study, moderate olive oil intake (Q2 vs. Q1: OR = 0.62; 95% CI: 0.39–0.99) and high intake (Q3 vs. Q1: OR = 0.47; 95% CI: 0.28–0.78) were associated with a lower risk of BC, corresponding to an approximate risk reduction of 38% and 50%, respectively. This protective association has been attributed to the antioxidant and anti-inflammatory properties of olive oil, particularly those related to its phenolic compounds, which may influence oxidative stress and cellular processes [38]. Furthermore, omega-3 fatty acids influence cell growth and enhance apoptosis. This inhibits angiogenesis, resulting in an anticancer effect [39]. Both the amount and type of fat are significant when evaluating the correlation between fats and BC. Diets high in monounsaturated and omega-3 fatty acids, while low in saturated and trans fatty acids, may diminish the risk of BC and potentially provide anticancer benefits by enhancing cellular defense mechanisms [12,37,39].

Preclinical evidence supports a plausible biological role of dietary fat types in BC-related mechanisms, whereas human evidence is largely observational. Therefore, the current findings do not establish causality and highlight the need for interventional studies.

#### 2.1.4. Proteins

Proteins are essential dietary components that, depending on their origin and metabolic impact, can significantly influence BC progression. They are broadly categorized as either animal- or plant-based, with each type having different biological effects on the bladder [40]. Observational human studies have reported that a higher consumption of animal-derived proteins, particularly red and processed meats, is associated with an increased risk of BC [12,41]. Several biological mechanisms have been suggested for clarifying this relationship. Firstly, cooking animal proteins at high temperatures leads to the production of carcinogenic heterocyclic amines (HCAs) and polycyclic aromatic hydrocarbons (PAHs). These compounds form reactive intermediates when metabolized in the body via phase I and phase II detoxification enzymes such as CYP1A2 and NAT2. These reactive metabolites can cause mutations and malignant transformation in bladder cells by forming DNA adducts (toxic compounds covalently bound to DNA). These cancer-causing substances have mutagenic effects on DNA [41,42]. Secondly, the iron present in red meat may enhance carcinogenic processes by promoting the synthesis of N-nitroso compounds (NOCs) [41]. Nitrosamines, especially those stemming from nitrate and nitrite derivatives present in processed meats, are excreted in the urine, thereby directly interacting with the bladder’s inner lining. This interaction initiates carcinogenic processes, including DNA alkylation, oxidative stress, and the disruption of apoptosis, as demonstrated in [41]. Furthermore, diets abundant in animal protein are also characterized by elevated levels of methionine and sulfur-containing amino acids. These components could potentially promote bladder carcinogenesis by inducing methylation imbalances, generating ROS, and instigating epigenetic alterations. Thirdly, elevated animal protein intake stimulates proliferation by augmenting IGF-1 levels. This inhibits apoptosis and promotes tumor progression [43]. A study found that a 50-gram increase in processed meat consumption increases the risk of BC by 20%. Nitrosamine metabolites in processed meat are the main risk factors [44]. Furthermore, high animal protein intake can increase the acidity of the urinary environment, thereby prolonging the solubility and contact time of potential carcinogens with the bladder epithelium. This situation, especially when combined with low fluid intake, increases the concentration of carcinogens in urine and may suppress DNA repair mechanisms [40].

Plant-based protein sources are reported to have protective effects against cancer. Grains and legumes are rich in phytochemicals, including isothiocyanates, flavones, and isoflavones. These phytochemicals can inhibit tumor development in the bladder mucosa due to their anti-inflammatory, anti-proliferative, and pro-apoptotic characteristics [45,46]. When consumed together with polyphenols and phytosterols, they suppress the expression of inflammation markers such as NF-κB, Cyclooxygenase 2 and inducible nitric oxide synthase, thereby reducing chronic inflammation and oxidative DNA damage. In addition, plant proteins neutralize urine pH by reducing urea and ammonia production, which is another protective mechanism that limits the contact of carcinogenic metabolites with urine [47]. One study found that a 3% increase in energy intake from animal protein was associated with a 15% increase in BC risk, while a 2% increase in energy intake from plant protein was associated with a 23% decrease in BC risk [48].

A large analysis of 10 prospective cohort studies (*n* = 434,412; mean follow-up 11.4 years) reported no significant association between total, animal-based, or plant-based protein intake and BC risk, and substituting 30 g/day of animal-based protein with plant-based protein did not influence BC risk [40]. In conclusion, while a potential correlation between BC and protein sources has been highlighted, additional research is required to elucidate their link.

### 2.2. Micronutrients

#### 2.2.1. Vitamins

Vitamins are necessary for crucial processes and critical for cellular metabolism and antioxidant defense in cancer metabolism. Vitamins A, C, D, and E and folate are notably significant in relation to BC. These vitamins are believed to influence bladder carcinogenesis directly or indirectly through their regulatory roles in DNA synthesis and repair, antioxidant capacity, and immune response [49,50,51,52].

Vitamin A (retinoids) and carotenoids have been associated with tumor-suppressive effects in BC. A retrospective study on BC showed that the risk increases in individuals with low vitamin A intake [53]. Retinoids inhibit proliferation and induce apoptosis by regulating gene expression via retinoic acid receptors [50,54]. They have considerable chemo-preventive and therapeutic potential for BC. In the early stages of this disease, the retinoic acid pathway is significantly disrupted, characterized by increased activity of STRA6 and SOX9 and suppression of regulatory genes such as FOXA1, PPAR, and RXRA. This imbalance can lead to problems in urothelial cells and signaling pathways and the development of abnormal apoptosis [54]. Mechanistic evidence further suggests that vitamin A may partially restore retinoic acid signaling by increasing the expression of LRAT and Neurod1, potentially reducing epithelial damage and abnormal urothelial cell proliferation [55]. Retinoids have also been reported to suppress epithelial–mesenchymal transition (EMT) through increased E-cadherin expression, a key process in BC development [54]. Moreover, the molecular changes often seen in BC, such as decreased LRAT and CRBP1, RORC suppression, and increased ALDH1A1/TUBB3, are also linked to retinoid resistance. Thus, vitamin A and retinoid-based interventions may help rebalance these pathways, potentially reducing tumor aggressiveness and improving therapeutic response. Carotenoids mitigate oxidative stress and DNA damage by their free radical scavenging capabilities [56]. Epidemiological studies indicate that diets high in carotenoids may reduce BC incidence; nevertheless, the results are notably inconsistent [57,58].

Vitamin C is a potent water-soluble antioxidant that actively mitigates reactive oxygen species and safeguards against DNA damage [23]. Continuous exposure of the bladder mucosa to carcinogens present in urine amplifies the significance of vitamin C in preserving epithelial integrity. Vitamin C may also reduce the formation of carcinogenic nitrosamines in the bladder mucosa, which is particularly relevant in experimental BC models induced by N-butyl-N-(4-hydroxybutyl)-nitrosamine [59,60,61]. In cohort studies, higher vitamin C intake has been associated with a lower risk of BC [52,62]. However, evidence from meta-analyses showed no significant association [59,61].

Recent studies have focused on the role of vitamin D in the mechanisms underlying multiple cancers, including BC. Vitamin D plays a role in the regulation of the cell cycle and apoptosis, and its active form, calcitriol, modulates gene expression via the VDR/RXR complex [49,63]. Consequently, vitamin D activates cyclin-dependent kinase inhibitors, such as p21 and p27, which in turn halts the cell cycle. Furthermore, it induces apoptosis by elevating the Bax/Bcl-2 ratio and suppresses EMT through increased E-cadherin expression. Thus, the uncontrolled proliferation and invasion of urothelial cells are limited. Vitamin D mitigates inflammation by modulating gene expression via the vitamin D receptor (VDR) while also inhibiting proliferation and increasing the efficacy of tumor-suppressor genes [63]. Beyond its direct cellular effects, vitamin D has been shown to influence the gut microbiota composition and immune homeostasis, thereby indirectly modulating systemic inflammatory responses. Such microbiome-mediated immune mechanisms may represent an additional pathway through which vitamin D status contributes to BC susceptibility and therapeutic response [64]. Evidence from meta-analyses and case–cohort studies investigating the association between vitamin D and cancer risk has yielded conflicting findings; some studies have reported an inverse association with higher serum vitamin D levels, whereas others have identified no significant association [59,65,66].

Vitamin E, due to its lipophilic form, safeguards unsaturated fatty acids in the cell membrane from oxidation, hence maintaining cell membrane integrity. Vitamin E serves as a significant antioxidant, mitigating oxidative stress pathways linked to carcinogenesis [67]. Moreover, vitamin E inhibits proliferation and promotes apoptosis by modulating signaling pathways, including protein kinase C, in cancer mechanisms [68]. In a randomized controlled trial, vitamin E supplementation showed inhibitory effects on bladder tumor development. Given that oxidative stress is known to promote cellular mutations and tumor formation in BC, these findings are considered biologically significant [69]. At the cellular level, vitamin E has been reported to modulate oxidative stress-related pathways, tumor-suppressor activity, and heat shock protein-mediated stress responses, thereby influencing cell cycle regulation and apoptosis [70,71]. A meta-analysis showed that vitamin E consumption is inversely proportional to the risk of BC and found that increasing vitamin E consumption to 50 mg/day reduced the risk of BC by approximately 4% [72].

B vitamins are crucial in inhibiting the transformation of normal cells into malignant cancer cells, as they participate in energy metabolism, mitigate oxidative stress, and modulate methylation [73]. In prospective cohort studies, the association between vitamin B1 intake and bladder cancer has been found to vary by sex. A marginal increase in BC risk has been observed among men with higher vitamin B1 intake, whereas an inverse association has been reported in women. This difference has been attributed to sex-specific dietary sources of vitamin B1, with men predominantly obtaining vitamin B1 from animal-based foods and women from plant-based sources [74]. Moreover, experimental studies indicate that vitamins B2 and B6 contribute to the mitigation of oxidative stress by catalyzing enzymes that govern DNA damage. A cohort study determined that elevated vitamin B6 consumption in women is inversely related to the incidence of BC [75].

Folate is a crucial vitamin for DNA synthesis and methylation. Folate deficiency is associated with DNA damage and genomic instability through aberrant methylation [76]. Recent investigations indicate that decreased folate levels may elevate the risk of BC [23,49]. The potential proliferative consequences of excessive folate supplementation are contentious, and the evidence is notably discordant [23,77].

Vitamin B_12_ plays a role in maintaining DNA integrity; however, while experimental evidence suggests that deficiency may mimic DNA damage [78], another study found no association between high vitamin B_12_ intake and BC risk [79]. Vitamin K has become a highly debated topic in recent years due to its potential anti-tumor effects related to cancer cell metabolism and cellular death pathways [80]. Preclinical studies suggest that vitamin K may induce metabolic stress and autophagy-related cell death through pathways involving the PI3K/Akt –HIF-1α axis and AMPK/mTORC1 signaling, although further studies are required to establish clinical relevance in BC [81].

Overall, mechanistic and preclinical studies support a biological role of several vitamins in BC-related pathways, whereas human evidence is largely observational and inconsistent. Therefore, current findings should be interpreted as hypothesis-generating, and further interventional studies are required to clarify clinical relevance.

#### 2.2.2. Minerals

Minerals are crucial for cellular homeostasis, DNA repair, and antioxidant protection. Studies in recent years have highlighted an association between selenium, zinc, calcium, sodium, iron, magnesium, and phosphorus and BC [82]. These minerals are believed to influence BC development through immunological modulation, antioxidant enzyme activity, and mineral-specific effects on cellular signaling, rather than through uniform cancer-related mechanisms [83,84,85].

Selenium is among the minerals most commonly linked to BC. Selenium, a trace element essential for numerous biological functions, including antioxidant defense, DNA repair, cell cycle regulation, and immune system modulation, primarily mediates its effects via selenoproteins. Meta-analyses have demonstrated that a higher selenium status is associated with an approximately 30–40% reduction in BC risk [86]. Selenium is a component of antioxidant enzymes such as glutathione peroxidase and thioredoxin reductase, which neutralize hydrogen peroxide and lipid peroxides, thereby limiting oxidative DNA damage [85,87]. Selenium promotes the eradication of cancerous cells via its apoptotic characteristics. Experimental research indicates that selenium can inhibit proliferation and induce death in bladder tumor cells [85,88]. Reduced serum selenium concentrations have been epidemiologically linked to an increased incidence of BC [86,89,90].

Zinc is an essential mineral involved in DNA repair, cellular development, and immune regulation; as a cofactor of superoxide dismutase, it reduces oxidative stress and supports genomic stability through tumor-suppressor pathways associated with DNA polymerase and p53. Zinc deficiency may increase the risk of carcinogenesis by promoting DNA damage [85,91,92]. In a BC cell line, treatment with zinc citrate resulted in a dose- and time-dependent reduction in cell number, accompanied by decreased mitochondrial aconitase activity, DNA fragmentation, increased caspase-3 activity, upregulation of p53 and p21, downregulation of anti-apoptotic Bcl-2/Bcl-xL, and increased pro-apoptotic Bax expression [93]. Another study found an inverse relationship between serum zinc levels and telomerase activity in BC patients. Patients with low serum zinc levels had higher telomerase activity [94]. Increased telomerase activity could contribute to cell immortality. This suggests that a lack of zinc might increase the risk of cancer by affecting how cells age and repair themselves. Epidemiological evidence regarding the relationship between zinc and BC is unclear; certain studies indicate that high intake may confer protection, but others find no significant correlation [84]. Emerging evidence suggests that trace elements such as selenium and zinc may also influence host–microbiome interactions by affecting epithelial barrier integrity and immune signaling pathways [95]. These effects highlight a potential indirect role of micronutrients in shaping the inflammatory and immune milieu relevant to bladder carcinogenesis.

Calcium acts as a second messenger in cellular signaling and may contribute to the regulation of proliferation, differentiation, and apoptosis [96,97]; however, excessive calcium intake may increase urinary calcium levels and promote crystal or stone formation, potentially contributing to bladder irritation [98]. The sustained presence of stones or calculi can cause chronic irritation and inflammation of the bladder mucosa; this state could be viewed as a potential risk factor, especially concerning BC. The preventive role of calcium in colorectal cancer is widely established, although research on its impact on BC remains scarce. The interplay between calcium and vitamin D metabolism complicates the evaluation of calcium’s possible impact on bladder carcinogenesis [23].

Sodium is an electrolyte that is very important for maintaining the equilibrium of fluids outside of cells. Nevertheless, excessive dietary sodium consumption can modify the urine composition, resulting in elevated sodium concentrations in urine and subsequent alterations in urine pH and osmolality [99]. A meta-analysis contrasting the highest and lowest sodium intake categories found that elevated sodium intake correlates with a 62% increased likelihood of BC (OR = 1.62; 95% CI: 1.04–2.55). Some researchers believe that high sodium intake may cause BC by increasing the growth rate of tumor cells, the strength of inflammatory reactions, and the amount of potential carcinogens that are discharged in urine [82].

Iron and magnesium are minerals that are essential for making and repairing DNA and for using energy [100]. A meta-analysis comparing the greatest and lowest intakes of iron or magnesium revealed no significant difference in the risk of BC between the categories [82]. Iron and magnesium minerals on their own do not seem to be important risk or protective factors in the development of BC. Nonetheless, some believe that low magnesium consumption and high calcium and phosphorus intake may be linked to an increased risk of BC [101]. This meta-analysis likewise identified no significant correlation between phosphorus intake and BC [82]. This scenario indicates that the potential influence of elevated phosphorus intake, diminished magnesium intake, and calcium mechanisms on BC may occur indirectly via mineral interactions. This could harm the integrity of the urothelium [101].

### 2.3. Phytochemicals

Phytochemicals are bioactive substances that are inherently present in plant diets and influence biological processes [102]. Recent experimental and epidemiological research has suggested possible preventive effects of phytochemicals against BC. These effects are attributed to the enhancement of antioxidant defenses, detoxification of carcinogens, regulation of the cell cycle, and activation of apoptosis. Phytochemicals exhibit distinct functions in the prevention and modulation of BC, diverging from the actions of conventional chemotherapeutic agents. Their application in chemoprevention is particularly noteworthy; chemoprevention is characterized by preventing, suppressing, or reversing the initial stages of carcinogenesis or by impeding the invasive capabilities of premalignant cells. Unlike treatments for advanced cancer, phytochemicals primarily target the control of carcinogenesis [103,104]. Many dietary phytochemicals are converted into biologically active metabolites by the gut microbiota. These microbiota-derived metabolites have been reported to exhibit immunomodulatory properties, influencing both systemic inflammatory status and local immune responses [105]. Therefore, it is suggested that some of the anticancer effects of phytochemicals may arise not only through direct cellular mechanisms but also via the diet–microbiome–immunity axis.

Mechanistic and preclinical studies indicate that polyphenols represent one of the most thoroughly investigated categories of phytochemicals in relation to BC. Compounds such as flavonoids, resveratrol, and quercetin, classified as polyphenols, mitigate DNA damage by neutralizing free radicals. These chemicals impede the cell cycle, restrict tumor cell proliferation, and activate apoptotic pathways [103]. Moreover, phytochemicals can induce anti-inflammatory actions inside the tumor microenvironment by obstructing signaling pathways, including NF-κB [103]. Additionally, it has been determined that polyphenols exhibit stronger inhibitory effects on the growth of T24 and J82 cells, which are human bladder urothelial carcinoma cells [106].

While some polyphenols have been shown to slow the growth of bladder tumors in lab and animal studies, others have been found to stop T24 BC cells in the G2/M phase. This occurs because cyclin B1 and cyclin-dependent kinase cell division control protein 2 homolog are reduced [107]. Moreover, phenols have exhibited inhibitory effects on the migration of BC cells by downregulating the expression of matrix metalloproteinase (MMP)-9 [108,109], a significant phytochemical that belongs to the flavanone class of polyphenols, has been identified as a novel MMP-2 inhibitor, and is potentially capable of suppressing BC metastasis by obstructing BC cell migration [23].

The relationship between cruciferous vegetables and BC has become a frequently researched topic. This is mainly because of their glucosinolate derivatives, namely, isothiocyanates, especially sulforaphane. A review of studies shows that sulforaphane activates the Keap1–Nrf2/ARE pathway, inducing phase II detoxification enzymes, suppressing phase I carcinogen-activating enzymes, and inducing cell cycle arrest and apoptosis in urothelial cells [110]. Experimental and observational studies indicate that lycopene, a carotenoid, may contribute to reducing the risk of BC by decreasing oxidative stress through its antioxidant activity and by modulating signaling pathways involved in cell proliferation and inflammation [111]. Phytochemicals serve a protective function through mechanisms including control of cell cycle progression, reduction in DNA damage, epithelial–mesenchymal transition (EMT), promotion of apoptosis, enhancement of carcinogen detoxification, and prevention of inflammation [103,104,112]. The number of clinical investigations is quite limited, and epidemiological and experimental results require validation through extensive human research [23,103,104].

### 2.4. UPFs

Recent years have seen a rise in nutritional studies investigating the health impacts of ultra-processed foods (UPFs) [108,109]. UPFs are defined by the incorporation of sugars, colorants, chemicals, and preservatives, emphasizing flavor and convenience at the expense of nutritional value [113,114]. They are often defined by elevated energy density and reduced fiber and antioxidant levels. Greater exposure to UPFs, expressed as each 10% increase in their contribution to dietary energy, has been associated with a higher risk of chronic diseases, including cancer [113]. Dietary patterns characterized by excessive consumption of processed meats, sugary foods, and refined products, common in Western diets, have been linked to a significantly increased risk of BC in extensive cohorts [115]. This correlation can be partially elucidated by the conversion of nitrite and nitrate additives in processed meats into N-nitroso compounds inside the urinary system, which have mutagenesis effects on the bladder epithelium [116,117]. Nitrosamines circulate in the bloodstream in a soluble form and are mostly removed from the body through urine. When these compounds are excreted in urine, they come into direct contact with the bladder lining. This contact can cause alkylation, mutations, and oxidative damage to DNA. As a result, these substances are thought to cause genetic changes in the cells of the bladder lining. This could disrupt the normal cell cycle and, over time, initiate the process of cancer development. Additionally, HCA and PAH chemicals generated during the high-temperature cooking of meats induce genetic harm by creating DNA adducts and promoting tumorigenesis [41]. The additives and packaging chemicals contained in UPFs may also be factors associated with BC. Chemicals found in packaging, such as bisphenol A (BPA) and phthalates, are endocrine disruptors that mimic the effects of estrogens and can stimulate signaling pathways in cells, such as estrogens receptors (ERs), estrogen-related receptor gamma and MAPK. Activation of these pathways may lead to increased proliferation of urothelial cells, facilitate cell migration and invasion, and accelerate EMT processes [23].

The sodium content in UPFs is also quite high [118]. The effect of sodium on BC is explained by several biological mechanisms. Firstly, high sodium intake may increase cell proliferation and inflammatory response in the bladder epithelium. Sodium salts have been experimentally identified as factors that promote tumor development [82]. Excess sodium alters urine composition, increasing the concentration of potential carcinogens in urine. This alters the pH of urine and paves the way for the formation of crystals and stones that damage the bladder epithelium [82,119]. In addition, high sodium consumption contributes to the carcinogenesis process through inflammation. Sodium disrupts intracellular ion balance and increases the production of inflammatory cytokines (e.g., IL-6 and TNF-α), leading to constant irritation and oxidative stress in the bladder mucosa [82]. This can cause DNA damage, cellular mutations, and ultimately tumor formation.

Excessive intake of sugary and refined meals, classified as ultra-processed, exacerbates chronic inflammation by promoting obesity and insulin resistance. Simultaneously, these products induce insulin secretion, resulting in hyperinsulinemia and elevated IGF-1 levels, which promote bladder epithelial growth. This may inhibit apoptosis [24,65,113]. The correlation between UPF consumption and BC risk can be elucidated through both direct (carcinogen production) and indirect (obesity and metabolic disturbances) mechanisms [24,41,113,116,117]. In addition, high consumption of ultra-processed foods has been associated with gut microbiota dysbiosis, reduced microbial diversity, and increased endotoxin translocation [120]. These alterations may promote chronic low-grade inflammation and adversely affect the bladder immune microenvironment, thereby contributing to bladder carcinogenesis through the diet–microbiome–immunity axis. Consequently, limiting UPFs, a significant component of the Western diet, may be regarded as a crucial dietary strategy for the prevention of BC [121]. A study has shown that adhering to a Western diet increases the risk of BC by 1.46 times. The Western diet consists of fried foods, meat and processed meat, which are positively associated with the recurrence of non-muscle-invasive BC [44].

### 2.5. Food Groups

Studying the impact of food groups on BC can enhance the understanding of carcinogenesis. Fruits and vegetables, whole grains, dairy products, and animal products have been extensively examined within this context [12,26,49,115,122].

Fruits and vegetables, abundant in vitamins, minerals, fiber, and phytochemicals, are recognized as food groups with an effective protective effect against BC [49]. The antioxidant chemicals included in these foods inhibit DNA damage by neutralizing free radicals, reducing inflammatory reactions, and restricting cellular proliferation [123]. Moreover, tumorigenesis can be inhibited by obstructing angiogenesis and promoting apoptosis. Vegetable and fruit consumption may increase the dilution or excretion of potential carcinogens in urine; this is significant considering that the bladder epithelium is more exposed to these substances via this route [14]. Many studies have reported that fruit and vegetable intake may reduce the risk of BC. The World Cancer Research Fund report also states that components found in fruit and vegetables, such as antioxidants, flavonoids, and dietary fiber, may have a direct effect on the bladder epithelium [124]. One study also found that a high intake of fruit and vegetables was associated with a 21% lower risk of BC.

Whole grains and fiber-dense diets reduce the absorption of carcinogens in the gastrointestinal tract and promote urine elimination of potentially harmful substances [26]. Moreover, mitigating hyperinsulinemia and IGF-1-driven proliferative signals by reducing the glycemic load serves as a protective mechanism against bladder carcinogenesis [125].

The association between dairy intake and BC risk is complex, as these products contain both potentially protective and risk-enhancing components. Certain cohort studies have indicated no substantial correlation between dairy intake and BC. Confounding variables, including gender and smoking, are believed to affect these outcomes [122,126]. However, the casein, lactose, vitamin A, calcium, and vitamin D in milk have a protective effect against many types of cancer, while the lactic acid bacteria in dairy products strengthen the body’s anticancer immunity. Additionally, the apoptotic and anti-proliferative effect of Conjugated Linoleic Acid (CLA) in milk may inhibit BC. Furthermore, CLA increases apoptosis in cancer cells and reduces cell proliferation by inhibiting the insulin-like growth factor receptor (IGF-IR). Nonetheless, the calcium and bioactive peptides in dairy products may confer protective effects; conversely, it has been proposed that the saturated fatty acid content may elevate risk [126,127,128].

The consumption of red and processed meat is regarded as a risk factor for BC. Nitrite and nitrate additives, especially in processed meats, are transformed into N-nitroso compounds upon contact with urine, having a carcinogenic effect on the bladder mucosa [41,115,117]. Moreover, it has been highlighted that heterocyclic amines (HCAs) and polycyclic aromatic hydrocarbons (PAHs), present in red meat cooked at elevated temperatures, induce genetic damage by creating DNA adducts and may expedite tumorigenesis. A high intake of saturated fat associated with red meat consumption is linked to increased inflammation and oxidative stress; chronic inflammation may contribute to cell proliferation, DNA damage, and the risk of oncogene activation [41,129]. A case–control study showed that consuming red meat at least five times a week resulted in twice the incidence of BC compared to consuming meat less than once a week [130]. Although the available evidence reveals consistent trends regarding the association between food groups and BC, how these trends translate into clinical decision-making remains unclear.

**Figure 1:**
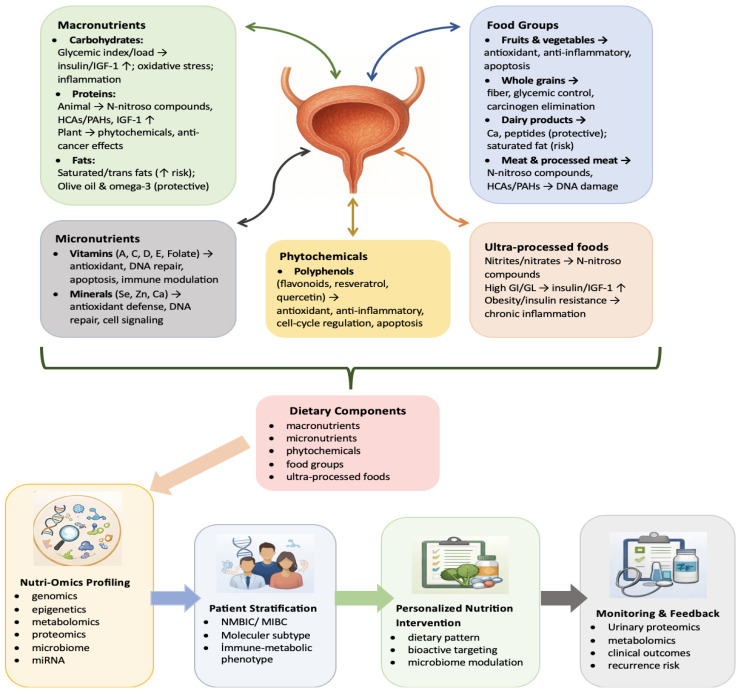
Mechanistic links between dietary components and bladder cancer biology and their integration into a precision nutrition workflow.

However, the interactions between nutrition and BC are often not linear and do not exhibit homogeneity across individuals. The observed variability arises from the convergence of multiple factors, including dietary patterns, dose and duration of intake, genetic background, metabolic phenotype, and environmental influences. This suggests that population-wide, uniform dietary recommendations may not adequately reflect biological responses or clinical outcomes. In particular, diet-induced alterations in the gut and urinary microbiota appear to play a decisive role in immune regulation, thereby influencing both BC-specific immune surveillance and systemic inflammatory status. This diet–microbiome–immunity axis provides a strong biological foundation for shifting from universal dietary recommendations toward individualized precision nutrition approaches in the prevention and risk management of BC. Figure 1 illustrates the mechanistic links between dietary components and BC biology and integrates these mechanisms into a precision nutrition workflow.

## 3. Precision Nutrition: Fundamental Principles and Applications in BC Prevention

Food safety and security, along with optimal nutritional patterns and sustainable consumption, have long been seen as key to improving health and treating non-communicable diseases. Adequate and balanced nutrition is one of the most important parts of a healthy and sustainable life [131]. In many countries nowadays, dietary advice is given based on nutrition guidelines designed to meet the needs of the general population [132]. However, variations in personal, metabolic, digestive, health, and illness factors may influence people’s nutritional requirements [133]. Precision nutrition plans that take into account a person’s entire medical background are required for long-term health, not general dietary suggestions [134]. Precision nutrition is characterized as a customized dietary strategy designed to fulfill an individual’s ideal requirements. Nutritional practices are characterized by an individual’s phenotype, nutrigenome, metabolome, epigenome, microbiome, proteome, and transcriptome data, alongside environmental factors, physical activity, lifestyle, psychosocial traits, eating attitudes and behaviors, and a comprehensive nutritional history [135,136]. Precision nutrition can go beyond the “one-size-fits-all” model and meet each person’s unique nutritional needs by taking into account the bigger picture of precision medicine [137]. Recent progress in omics technologies, computational biology, and data science has accelerated the adoption of this method and opened up new ways to stop nutrition-related chronic diseases like obesity, type 2 diabetes, heart disease, and some cancers [138]. In this way, nutritional science is expected to continue to play a large role in treating a growing number of metabolic disorders in the next few years. This will provide the best nutritional management for preventing noncommunicable chronic diseases and protecting public health in the long term [139,140]. The goal of providing precision dietary advice is to encourage people to make adjustments to their routine and habits that will benefit their health in the long run [141].

All individual sources of data for precision nutrition planning are deemed adequate if they are evidence-based and scientific, provide supported scientific evidence for the designated tool, and are widely accepted. The attributes of the required evidence may differ based on the prospective advantages, hazards, and individual variances of the relevant variables [142]. Precision nutrition planning is crucial for individuals to sustain dietary and lifestyle behavior changes, providing both protective and therapeutic benefits against cancers like BC, and ensuring a sustainable quality of life during and after treatment [143]. With this harmony, the individual’s sustained personal and social health benefits should be quantified using methodologies and metrics, and “phenotypic flexibility,” the ability to adjust to changing conditions, should be shown. Protective and adjuvant treatments for all cancers, including BC (considering complications, weight loss, and hospital stay), should be developed [144,145]. A study reported that precision dietary planning plus nutrition education, pharmacotherapy, motivational interviewing, and cognitive–behavioral therapy helped improve cancer symptoms before or during cancer treatment [146]. In another multi-component study that evaluated changes in body weight or composition, as well as changes in nutritional status, it was reported that the use of precision nutrition or dietary plans and individual recipes, combined with additional interventions such as training or counselling, was effective across different cancer types and cancer treatments [147]. In a randomized pilot trial conducted on patients with non-invasive BC (*age*: 50–80 and *n*: 48), the behavioral goals of the intervention were set to encourage the consumption of seven servings of vegetables per day, with at least two of them being cruciferous vegetables, as part of a precision nutrition plan. The results showed that the precision nutrition plan induced dietary changes associated with protective effects against BC in patients with non-invasive BC. This data demonstrates the feasibility of implementing therapeutic dietary changes to prevent recurrent and progressive BC [148].

The lack of sample availability, intratumoral heterogeneity, and established models for disease progression has been a key challenge in elucidating the functional impact of genomic alterations in urological cancers, both in their common and rare forms [149]. In the context of precision medicine, it is extremely valuable to characterize tumor heterogeneity from a metabolic perspective. The modulation of metabolic levels, providing a combinatorial approach with therapeutic agents, is of great importance for the sustainable treatment of the disease [150]. However, despite the continued lack of information about the metabolome, it has been possible to identify subtypes of urological cancers, particularly those of the prostate and bladder, based on genomic changes [149]. Although human genomes exhibit 99.9% similarity, the 0.1% variation is the consequential factor that makes each individual distinct [151]. In recent years, advanced methodologies linked to nutrition and food science technology have focused on understanding individual variability in response to nutrients to provide precision nutrition recommendations for patient subgroups [152]. Nutrigenomics, nutrigenetics, and phenotypic variables can influence how individuals respond to diet, nutrients, metabolic activity, and treatment outcomes [153]. A key way to figure out how the body reacts to different foods and develop a personalized diet plan is to cross-map a person’s genetic and metabolic profiles [154]. Nutrigenetics can utilize many methods developed in the fields of nutrition and genomics, including high-throughput omics technologies such as nutriomics (genomics, epigenetics, transcriptomics, proteomics, microbiome, and metabolomics) and metagenomics [155]. A precision nutrigenetic profile is created by providing information on how an individual’s genes or body respond to the nutrients that they consume [156]. Recent studies have shown that many genomic markers, including copy number variations, epigenetic markers, and single-nucleotide polymorphisms, influence how our bodies react to different types of nutrients [157,158]. However, the absorption and metabolism of nutrients, molecular-level enzyme interactions with nutrient cofactors, and health effects are dependent on inherited genetic variants, which can alter biochemical reactions. The health benefits can be maximized by optimizing nutrient profiles and needs to match an individual’s genetic characteristics and by considering their lifestyle, nutritional profile, and health status [159]. Precision nutrition optimization also takes epigenetics into account. It lays the groundwork for nutrigenomics and its use in individualized nutrition by concentrating on how modifications to proteins and DNA, interactions between histones and DNA, DNA methylation, and chromatin structure can take place without changing nucleotide sequences [160]. Among the many epigenetic mechanisms are the following: DNA methylation and hydroxymethylation, which control gene expression levels; acetylation, methylation, phosphorylation, ubiquitination, and sumoylation, which control chromatin structure; and the regulation of gene expression levels through short and long non-coding RNA molecules (ncRNAs) [161]. Dysregulated epigenetic mechanisms play a role in various diseases, including cardiovascular disorders, neurodegenerative diseases, obesity, and particularly cancer [162]. The transcription and translation of genes can be altered by macronutrients, micronutrients, vitamins, minerals, and phytochemicals, which affect homeostatic responses such as metabolism and cell growth, which are important in disease progression [163]. Dietary phytochemicals, including food-derived flavonoids, as well as tetraterpenoids, organosulfur compounds, and isothiocyanates, such as genistein, resveratrol, curcumin, and polyphenols found in vegetables, fruits, medicinal plants, and beverages, can modulate the activity of transcriptomic responses and changes in chromatin structure through epigenetic modulation mechanisms, and they exhibit various biological effects such as anticancer, antioxidant, and immunomodulatory properties [162]. In a previous study, phytochemicals such as curcumin, epigallocatechin-3-gallate, genistein, quercetin, resveratrol, and sulforaphane showed promising results in regulating cancer-related epigenetic processes, such as DNA methylation, histone modification, and non-coding RNA regulation, and cancer-protective effects [164]. Endogenous microRNAs derived from food have the potential to regulate the gut microbiota, interact with cells in the gastrointestinal system, and, after absorption in the intestines or stomach, reach target organs; this makes them an important nutrigenomic player in cancer prevention and human health regulation [165]. Within the realm of precision nutrition, miRNAs possess significant potential as biomarkers for monitoring and predicting individual responses to dietary changes [166]. A systematic review examined the influence of food and specific dietary components on miRNA expression, identifying a total of 108 miRNAs regulated by dietary factors. Dietary habits are confirmed to be intimately associated with the modification of endogenous miRNAs [167]. A study investigating bioactive dietary components that modulate miRNA expression revealed that curcumin, resveratrol, quercetin, ω-3 fatty acids, and fiber influence pathways including cell cycle arrest, invasion, metastasis, apoptosis, cell proliferation, cell growth, angiogenesis, inflammation, chemoprevention, tumor growth, and plasminogen activation by regulating specific miRNAs, thereby enhancing the efficacy of cancer treatment and aiding in cancer prevention [168]. Dietary bioactive compounds modulating cancer-related miRNAs and pathways with potential relevance for precision nutrition in BC are presented in Table 1. Significantly, the majority of diet–miRNA mechanistic associations outlined in Table 1 are based on preclinical evidence, and their clinical applicability in BC is still under investigation, necessitating validation through rigorously designed human studies.

Precision nutrition, as an individual-specific epigenetic diet, is considered the future of “precision medicine” for treating non-communicable diseases that may occur with aging and lifestyle, and for maintaining an individual’s optimal well-being [191]. Epigenetic mechanisms can directly modulate the expression of proto-oncogenes and tumor-suppressor genes, frequently manifesting in the initial phases of tumorigenesis and predominantly preceding genetic alterations. These characteristics render epigenetic targets exceptionally promising for cancer prevention and therapy [192]. In summary, dietary patterns, models, and components developed through precision nutrition interventions can function as activators, inhibitors, or substrates for epigenetic enzymes, thereby establishing a connection between nutrition and epigenetic regulation, and potentially offering protective effects against cancer or enhancing the quality of life of individuals during and after treatment [136,193].

The human microbiome, a dynamic ecology within the gastrointestinal tract, significantly influences general health. The interplay of nutrients in the gut orchestrates a complicated system that affects digestive functions and vulnerability to gastrointestinal illnesses [194]. The features of the gut microbiota can differ markedly among individuals based on their genetic profiles, lifestyles, and behaviors [195]. Strategies for improving gut microbiota through food, precision nutrition, and clinical indications can provide potential methods for managing gastrointestinal homeostasis and transforming sustainable healthcare [194]. Research indicates that macronutrients, including carbs, proteins, and fats, together with micronutrients, significantly influence the makeup and diversity of the gut microbiota [196]. Additional data associates alterations in the microbiota composition with specific inherited and immutable human traits, including ethnicity and geographic location [197]. The gut microbiome comprises bacteria that synthesize numerous vital metabolic compounds, including short-chain fatty acids (SCFAs) like acetate, propionate, and butyrate; amino acid metabolites such as ammonia and indole; essential vitamins, particularly K and B vitamins; postbiotics, including extracellular polysaccharides; fermentation metabolites like lactate and glycerol; and various bioactive components, including bacteriocins. Bacterial metabolites profoundly influence human health through several mechanisms, particularly through the regulation of metabolism (lipid, carbohydrate, and protein), preservation of intestinal barrier integrity, modulation of inflammation, and regulation of immunological responses [198]. Recent studies suggest that the composition of the gut microbiota can influence the body’s response to different diets, nutrient absorption, and metabolism [199,200]. Growing evidence indicates that the structure of the gut microbiota influences cancer development and susceptibility, and that nutritional factors can modulate these processes by promoting the growth of bacteria with tumor-suppressive or carcinogenic properties, depending on nutritional requirements. Furthermore, it has been reported that some dietary components can be metabolized by commensal or symbiotic gut bacteria into bioactive compounds with anticancer properties [201,202]. The accompanying investigation demonstrated that the urine microbiota influences the etiology, diagnosis, and treatment response of BC. Research indicates that microbial diversity and composition vary between patients with BC and healthy persons. Comparative analyses of tumor tissue and urine samples have shown unique microbial signatures. The microbiota associated with tumors demonstrates superior microbial diversity relative to urine samples. It has been reported that microbial populations in tissue samples may provide greater biomarker potential than those in urine samples [203]. A study revealed a significant causal relationship between the gut microbiota and BC, specifically showing that Bilophila is a significant pathogenic initiator [204]. Dietary habits can affect the gut microbiota, demonstrating the microbiome’s significance as a crucial component in precision nutrition. Consequently, variations among people and populations can influence the overall dietary response in terms of digestion, nutritional advantages, and customization [143]. The gut microbiota contains both environmental and host-specific information that could enable individualized nutrition and lifestyle recommendations; however, this data currently lacks the reliability needed for conclusive guidance. Consequently, dietary guidelines derived from individual microbiome composition analysis for typically healthy persons may not be suitable unless corroborated by recognized blood and physiological biomarkers [205]. The current distribution of the eubiotic microbiota composition is still ambiguous; however, ascertaining an individual’s microbiome composition could serve as a significant tool for precision nutrition, complementing existing personal data derived from emerging glycomic biomarkers in blood, as well as various genomes, epigenomes, metabolomes, and indicators of health and longevity. Nonetheless, the interpretation of individual microbiomes for tailored recommendations poses a challenge until microbiota relationships are reliably replicated and additional longitudinal cohort methodologies, such as randomized clinical trials and studies illustrating the causal effects of microbiome modification on health (beyond clinical) phenotypes, are undertaken [205]. A study demonstrated that a diet intervention based on precision nutrition could improve the gut microbiota (e.g., *Akkermansia muciniphila*) and levels of microbial metabolites (SCFAs and tryptophan derivatives), thereby boosting immunity. It also showed that this intervention was effective in cancer treatment when used as an adjuvant therapy with probiotics and postbiotics [206]. Precision dietary patterns that includes prebiotics, probiotics, synbiotics (a combination of prebiotics and probiotics), and postbiotics—all part of precision nutrition—were shown to enhance cellular and humoral immune responses to immunotherapy in another study [207]. Metabolites and the human microbiome interact dynamically, with the microbiome having a substantial impact on the metabolome in both the proximal and distal parts of the body, and the metabolome having an effect on the microbiota [208]. Overall, these observations suggest that specific microbial diets, including probiotics and postbiotics, have the potential to regulate cancer progression and treatment response, warranting further investigation in future studies [206]. In summary, the growing evidence concerning the gut microbiota’s mechanism of action in cancer initiation and progression, as well as its influence on anticancer therapies, will lead to significant changes in treatment efficacy for cancer patients, enhance prognosis, and expedite the advancement of precision medicine alongside precision nutrition [209].

Metabolomics include metabolites, which are diminutive molecules generated during biological activities across many organisms. Metabolic anomalies can result in the malfunctioning of metabolic pathways and the accumulation or lack of metabolites, which are recognized as distinguishing characteristics of diseases [210]. Metabolomics profiling has the ability to identify changes in small metabolites in response to environmental changes, including diet [211]. By analyzing all metabolites, a clear picture of metabolism and the molecular fingerprint can be obtained [212]. Characterizing food-derived biomarkers helps elucidate inter-individual variability in nutrition metabolism across health and disease states. Metabolomics research can enhance nutritional assessment in three primary ways: by discovering biomarkers linked to food intake, by examining diet-related disorders through cohort studies, and by assessing dietary treatments via metabolic patterns [213]. Such characterization can serve as a biomarker or an indicator of an organism’s biological state [212]. Substantial progress in science and technology over the last thirty years has led to the emergence of multi-omics, enabling the examination of cellular components that most significantly influence the final phenotype, despite metabolomics being less advanced than genomics, transcriptomics, and proteomics [214]. Recent advancements are encouraging for the application of omics technology in the progression of precision nutrition. This is notably intriguing since it can gather data on metabolic metrics, dietary consumption, concentrations of bioactive substances, and the impact of diets on endogenous metabolism. These elements encompass valuable insights for precision nutrition. The utilization of metabolomic profiles to delineate subgroups or metabotypes is essential for delivering tailored dietary recommendations [215]. Dietary biomarkers are metabolites that objectively indicate the intake of specific nutrients or dietary patterns and can be classified according to the level of evidence. Currently, there are several validated biomarkers of nutrient intake, such as alkylresorcinols, proline, betaine, and 3-carboxy-4-methyl-5-propyl-2-furanpropanoic acid [216]. Changes in plasma metabolite levels can result from changes in nutrient composition and can affect metabolite concentrations in the tumor microenvironment in BC. These changes can subsequently regulate cancer cell metabolism and therapeutic responses. Moreover, monitoring dietary factors through metabolomics to observe their impact on changes in diet-related cancer cell metabolism and metabolic weaknesses can create synergy with new or existing treatments or provide sustainable benefits [217]. Nonetheless, omics biomarkers reflecting the intake of dietary patterns, including a healthy plant-based diet, a healthy Nordic diet, Dietary Approaches to Stop Hypertension, the Mediterranean diet, a sugar-rich diet, UPFs, and a vegan diet, have been reported, with the majority requiring validation [218]. With this, precision nutrition, supported by nutrigenomic technologies, has revolutionary potential in individual health management. It is believed that the integration of genomic, epigenetic, transcriptomic, proteomic, microbiome, and metabolomic data, along with big data management and machine learning (i.e., through machine learning algorithms and/or artificial intelligence), will make it possible to develop specific, effective, and sustainable nutrition education modules, and will reveal important developments in the treatment of BC [23,136,219].

**Figure 2:**
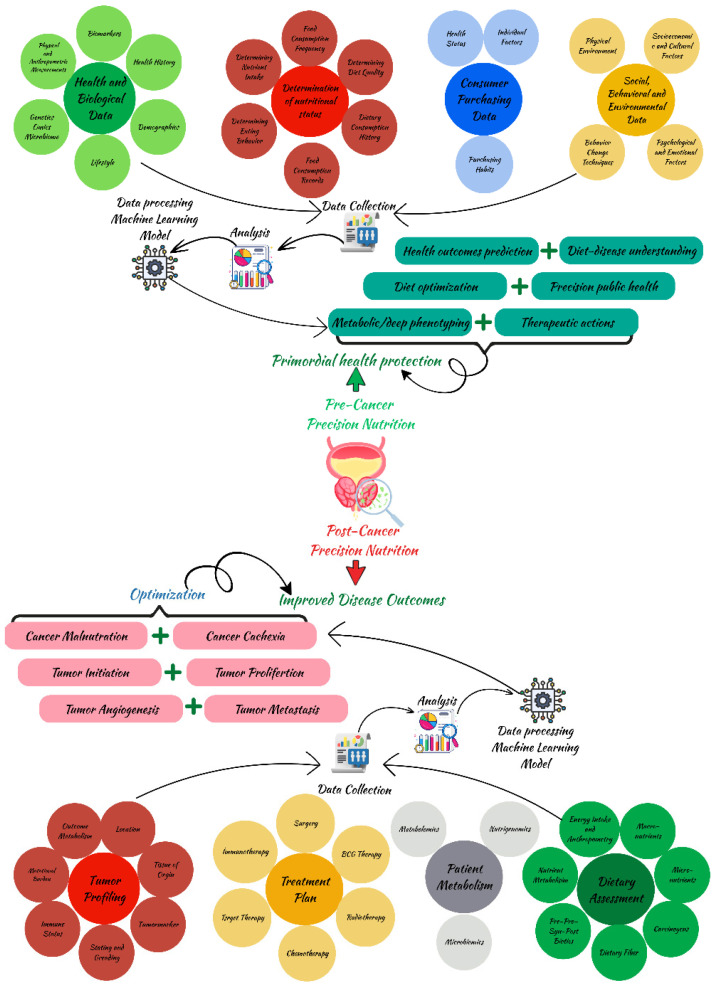
The importance of precision nutrition in the management of pre- and post-BC.

In conclusion, nutrition is important not only in the prevention of cancer but also in its progression and treatment, with one-third of cases characterized by malnutrition due to inadequate and unbalanced nutrition, impacting both the prevention of cancer and malignant conditions resulting from the disease [220]. By evaluating nutritional status for each cancer type, considering preventive and specific postoperative risks, and employing a precision nutrition approach, tailored nutritional dietary patterns and models can be created for individuals based on their unique conditions, adverse effects can be mitigated, and treatment efficacy can be enhanced [221]. The survival and quality of life of BC patients depend on early evaluation of their nutritional condition, creation of a personalized treatment plan using precision nutrition, and ongoing monitoring. This is because people’s lives and well-being depend on precision nutrition education, which is accessible through open educational resources in suitable settings and is easy to grasp and implement [220]. The importance of precision nutrition in the pre- and post-BC period is presented in Figure 2.

Studies have reported that BC patients with malnutrition or nutritional risk before surgery showed lower postoperative quality of life, higher complication rates, and significantly lower survival rates compared to well-nourished patients [222,223].

## 4. Precision Nutrition-Based Diets in BC Treatment

The rise of precision medicine in cancer treatment has led to the development of individualized treatment and dietary plans, which are based on the molecular causes of the disease. An increasing understanding of cancer biology has indicated that nutrition may serve not only as a contributing element in tumorigenesis but also as a direct therapeutic target [224]. Due to its high rate of recurrence, its complex biological structure, and its sensitivity to environmental factors, BC has led to a growing interest in lifestyle changes, alongside standard medical treatments [225]. The precision nutrition strategy seeks to create precision diets via the examination of the genetic, epigenetic, and metabolic profiles and microbiota composition of people [136]. Certain genetic variations can influence how individuals react to environmental carcinogens and dietary elements; consequently, a particular food item might confer protective benefits in one patient population while proving detrimental in another [226]. Recent findings from preclinical studies and initial clinical trials have revealed that specific dietary patterns significantly contribute to cancer prevention and treatment by inhibiting tumor formation, impeding tumor progression, and enhancing the efficacy of various anticancer therapies [227,228]. Furthermore, the impact of diet on BC extends beyond mere disease prevention; it has also been shown to modulate immune responses, inflammation, oxidative stress, and drug metabolism during therapeutic interventions [23]. Recent important nutritional models in the setting of cancer include calorie restriction, the ketogenic diet, intermittent fasting, the fasting-mimicking diet, amino acid alterations, and different dietary strategies [229].

### 4.1. Precision Recommendations for High-Risk Individuals

Nutritional interventions have gained significance in preventive oncology for individuals at elevated risk of BC, including smokers, individuals exposed to occupational carcinogens, those with a history of chronic inflammatory bladder diseases, or those with a genetic predisposition [23,230,231,232]. Precision nutrition techniques aim to address the molecular mechanisms that increase susceptibility to cancer [226]. For persons susceptible to nitrosamine metabolism, limiting processed meats and food containing nitrates/nitrites might mitigate mutagenesis effects on DNA [117]. Individuals carrying polymorphisms in phase I–II metabolism enzymes such as NAT2, GSTM1, and GSTT1, which are involved in detoxification processes, may detoxify environmental carcinogens more slowly. Individuals with higher susceptibility to oxidative stress may benefit from increased intake of fruits and vegetables rich in vitamins C and E, as well as polyphenols, which might protect the bladder mucosa by mitigating free radical damage [231,233]. For people susceptible to obesity and insulin resistance, selecting low-glycemic, fiber-dense whole grains may mitigate hyperinsulinemia and IGF-1-mediated proliferative signals [234].

For those at elevated risk stemming from chronic inflammation, dietary approaches aimed at mitigating inflammatory cytokines, such as tumor necrosis factor-alpha (TNF-α) and interleukin-6 (IL-6), are of considerable significance. The ability of omega-3 fatty acids to resolve inflammation via the synthesis of resolvins and protectins [235] could provide a crucial biological pathway for reducing proliferation and cellular stress within the bladder mucosa. Moreover, a diet abundant in fiber modulates both intestinal barrier integrity and systemic inflammation by augmenting the production of short-chain fatty acids, especially butyrate; this, in turn, fosters a less pro-inflammatory tumor microenvironment in individuals at high risk [27].

Variations in microbiota must be taken into account when formulating specific nutrition recommendations. In individuals with dysbiosis, bacteria that increase carcinogenic metabolites may be predominant. A diet rich in fiber, prebiotics, and probiotics mitigates systemic inflammation and enhances immune responses by augmenting the variety of the gut microbiota. Lactobacillus and Bifidobacterium species, in particular, can suppress inflammatory pathways that support tumor development by modulating T-cell responses [236]. This method may restrict the inhibitory effects of the tumor microenvironment in high-risk people [209].

### 4.2. Nutritional Support in Patients Receiving Chemotherapy/Immunotherapy

Chemotherapy and immunotherapy, often used to treat BC, can cause changes that negatively affect a patient’s nutrition. These changes include significant metabolic stress, increased inflammation, a loss of appetite, and the breakdown of muscle tissue [237,238]. Nutritional support throughout therapy is essential for preserving the patient’s overall health and improving treatment efficacy [145,237]. Recent studies indicate that patients with BC are at higher risk of malnutrition and sarcopenia after chemotherapy. This increases the likelihood of surgical complications, treatment side effects, and death [23,239].

Nausea, vomiting, mucositis, anorexia, and alterations in taste seen during chemotherapy adversely affect nutritional intake [238]. The systemic inflammation induced by chemotherapy elevates cytokine levels, thereby intensifying the acute-phase response within the liver and accelerating proteolysis in skeletal muscle. This catabolic state can swiftly lead to sarcopenia, particularly when protein and energy intake is insufficient. Consequently, the consumption of high-quality protein during treatment facilitates muscle protein synthesis, which in turn aids in preserving functional capacity and maintaining the intensity of the treatment regimen [239,240]. Insufficient protein and calorie consumption during this period elevates the risk of sarcopenia and malnutrition, thereby reducing treatment tolerance. Consequently, sufficient protein supplementation is crucial for preserving muscle mass [240]. Moreover, elevated oxidative stress resulting from treatment induces DNA damage and cellular apoptosis. Consuming fruits and vegetables abundant in antioxidant vitamins (C and E), polyphenols, and carotenoids helps safeguard normal cells by regulating reactive oxygen species (ROS) [241,242,243].

Nutritional interventions for BC patients undergoing immunotherapy are becoming increasingly significant due to various underlying processes. The primary objective of nutritional support for these patients is to bolster the immune system’s effectiveness. Recent research has revealed that the gut microbiota significantly influences the efficacy of anti- programmed cell death 1 (PD-1) and anti-PD-L1 therapies [244,245,246]. Specifically, the presence of certain bacterial species, such as *Akkermansia muciniphila*, and a higher concentration of short-chain fatty acid-producing bacteria may positively affect the immunotherapy response by augmenting CD8^+^ T-cell activity [246]. Fiber-rich diets improve immune cell activity and T-cell responses by augmenting the variety of the gut microbiota. Prebiotics and probiotics that affect the microbiome have been found to enhance the efficacy of immunotherapy [226,236]. Amino acids like arginine are crucial for T-cell proliferation and cytotoxic activity, and their adequate consumption may enhance the effectiveness of immunotherapy [23].

Nutritional support can enhance therapy efficacy by mitigating inflammation. Omega-3 fatty acid-rich diets diminish prostaglandin and cytokine synthesis, therefore mitigating immune suppression within the tumor microenvironment and enhancing the effectiveness of immunotherapies [23,247]. Likewise, specific dietary strategies (e.g., methionine restriction) can inhibit tumor cell proliferation while augmenting immune system efficacy [227]. Nutritional therapies serve as significant adjunctive measures to alleviate side effects, preserve quality of life, and enhance treatment efficacy [237]. This approach offers a comprehensive clinical framework, which helps improve treatment success and the overall well-being of people with BC.

### 4.3. Ketogenic Diet

The ketogenic diet is a dietary strategy characterized by a significant reduction in carbohydrate consumption, with energy predominantly sourced from fats. In this context, ketone bodies (β-hydroxybutyrate, acetoacetate, and acetone) serve as the principal energy source for the organism [248]. Normal cells efficiently utilize this metabolic fuel, but rapidly reproducing cells, such as BC cells, are predominantly reliant on glycolysis for energy production. The biochemical and cellular processes underlying the connection between BC and the ketogenic diet are the primary focus of current research. By restricting the metabolic adaptability of tumor cells, the ketogenic diet induces an energy deficit [19]. This dietary strategy may attenuate the Warburg effect by reducing glucose-to-lactate metabolic flux. This metabolic shift is accompanied by an upregulation of enzymes and glucose transporters, including HIF-1α, GLUT1, HK2, PFK, and LDH-A. As a result, the tumor cells’ dependence on glucose intensifies, leading to increased lactate production and the development of acidosis within the tumor microenvironment. Furthermore, lactate accumulation promotes both angiogenesis and immune suppression, thereby facilitating the tumor’s capacity for growth and dissemination [19,249].

Restricting energy metabolism elevates mitochondrial reactive oxygen species in cancer cells, resulting in DNA damage and apoptosis [19]. Conversely, ketone bodies enhance natural antioxidant defenses in healthy cells and offer targeted protection against oxidative stress. Moreover, the ketogenic diet inhibits the PI3K/Akt/mTOR signaling pathway by lowering insulin and IGF-1 concentrations. This route is a crucial mechanism promoting proliferation and tumor advancement in BC cells. The ketogenic diet restricts cellular proliferation by inhibiting this signaling network [19,227,250]. This strategy focuses on the reliance of BC cells on glycolysis for energy, thereby inducing “energy stress” within the tumor. A decrease in glucose availability subsequently reduces lactate production; consequently, lactate-mediated immune suppression within the tumor microenvironment could be reduced, potentially fostering a more conducive environment for T-lymphocyte activity. Furthermore, carbohydrate restriction might also impede the activation of glycolytic enzymes through HIF-1α; thus, metabolic factors that promote angiogenesis and invasion could be reduced.

Inflammation plays a significant role in the initiation and advancement of BC. The impacts of a ketogenic diet extend beyond metabolic functions. β-hydroxybutyrate inhibits the synthesis of inflammatory cytokines (TNF-α, IL-6, and IL-1β) and reduces the inflammatory response within the tumor microenvironment, thereby inhibiting the activation of the NLRP3 inflammasome [251,252]. It stimulates CD8+ cytotoxic T-cell activity and reduces regulatory T (T-reg) cells, resulting in a synergistic effect that may improve the efficacy of immunotherapy. Ketogenic diets offer a potential means of circumventing several immune evasion strategies by downregulating the expression of immune checkpoints CTLA-4 and PD-1 on tumor-infiltrating lymphocytes while simultaneously augmenting cytokine production and T-cell-mediated cytotoxicity [253]. Although these specific mechanisms have yet to be investigated within the context of BC, epigenetic alterations are recognized as significant contributors to tumor advancement and therapeutic outcomes; consequently, the epigenetic impacts of β-hydroxybutyrate could, in theory, modulate the underlying biology of BC. Preclinical evidence indicates that the ketogenic diet may effectively target cancer cells [254,255]. Furthermore, application of the ketogenic diet in BC treatment requires careful evaluation, considering the common clinical issues seen in patients, such as kidney damage, the risk of kidney stones, and muscle loss.

Therefore, the ketogenic diet might affect BC through several mechanisms. These include reducing the need for glycolysis, blocking the insulin/IGF-1/PI3K/Akt/mTOR pathway, increasing AMPK activation, reducing inflammasome activity, decreasing lactic acidosis in the tumor environment, and improving T-lymphocyte function. However, the variability in how ketones are metabolized, depending on the specific tumor type, and the potential for β-hydroxybutyrate to promote metastasis suggest that the ketogenic diet should be carefully and individually evaluated in BC patients. While the general mechanisms seem biologically relevant, there is still limited clinical evidence specifically related to BC. Nonetheless, clinical investigations are insufficient, and randomized controlled trials are necessary to clarify safety, sustainability, and efficacy [256].

### 4.4. Intermittent Fasting

Intermittent fasting is a dietary plan that alternates between periods of consumption and abstention at regular intervals. These intervals may include time-restricted eating patterns like 16:8 or 5:2, or extended fasting durations [257]. During fasting, cells adapt to the food deficiency by initiating defensive mechanisms, deriving energy from fatty acids and subsequently ketone bodies rather than glucose. This metabolic transition enhances DNA repair, stress resilience, and autophagy in normal cells, whereas in glycolysis-dependent cancer cells, it induces energy deficit and, subsequently, metabolic stress [258]. Autophagy transpires due to metabolic stress. Increased autophagy enhances cellular homeostasis by eliminating damaged organelles and proteins; however, in tumor cells, it reduces proliferative capacity and promotes death under nutritional deprivation [258,259]. The BC microenvironment is marked by persistent inflammation, elevated insulin levels, IGF-1 activation, oxidative stress, and metabolic irregularities. Consequently, the influence of intermittent fasting (IF) on these biological processes could potentially establish protective or progression-inhibiting mechanisms within BC.

Metabolic syndrome, obesity, and diabetes represent some of the most significant epidemiological risk factors for BC [260,261]. Conversely, intermittent fasting has the capacity to mitigate metabolic syndrome and hyperinsulinemia, as evidenced by its effects on weight reduction, enhanced insulin sensitivity, and decreased fasting insulin and IGF-1 concentrations [262]. Intermittent fasting induces metabolic adaptations primarily through periodic energy deprivation, leading to enhanced cellular energy sensing and activation of AMPK. Suppression of mTOR activity limits cellular growth and proliferation, while heightened AMPK activation promotes catabolic processes and metabolic flexibility [258,259,263]. Through these coordinated adaptations, intermittent fasting may attenuate tumor-promoting metabolic signals linked to dysregulated insulin/IGF-1 signaling and downstream PI3K/Akt/mTOR activity—pathways closely associated with obesity, insulin resistance, and diabetes—thereby contributing to a more favorable metabolic environment for BC.

Chronic inflammation represents another critical facet of BC. The urinary system continuously exposes the bladder mucosa to carcinogens via urine. This exposure activates NF-κB, which in turn promotes the upregulation of inflammatory cytokines (TNF-α and IL-6), as well as cell proliferation, angiogenesis, and treatment resistance [264]. In intermittent fasting, a reduction in NF-κB signaling and related systemic inflammation markers has been observed during the fasting phase [262]. Consequently, intermittent fasting may reduce the chronic inflammatory microenvironment within the bladder mucosa. Additionally, the synthesis of ketone bodies during fasting raises ROS levels, increasing oxidative stress and DNA damage in tumor cells, although antioxidant defenses are enhanced in normal cells [265]. Experimental models indicate that intermittent fasting may inhibit tumor growth, reduce metastatic potential, and improve responses to immunotherapies [266].

In summary, an assessment of the correlation between intermittent fasting and BC suggests that IF could potentially reduce the proliferative signals often present in bladder tumors. This effect is likely mediated through the suppression of the insulin/IGF-1/PI3K- Akt-mTOR pathway. Furthermore, IF might mitigate the carcinogenic microenvironment within the bladder mucosa by reducing NF-κB-driven chronic inflammation and proinflammatory cytokine levels. Consequently, it may demonstrate tumor-suppressive properties during the initial stages of cancer development by modulating oxidative stress. Nonetheless, research on the impact of intermittent fasting on BC is scarce; the duration and frequency of protocols, along with individual metabolic variations, may affect results. The hazards of extended fasting, including malnutrition, sarcopenia, and reduced treatment tolerance, must not be ignored [267].

### 4.5. Fasting-Mimicking Diet (FMD)

A fasting-mimicking diet is a low-calorie, low-protein diet that replicates the biochemical consequences of fasting while preserving important micronutrients [265]. This analysis examines how the fasting-mimicking diet (FMD) might work in BC. It focuses on how the diet changes metabolism, inflammation, and immune responses, and how these changes relate to known molecular processes in bladder tumors.

During the fasting-mimicking diet (FMD), insulin-like growth factor 1 (IGF-1) levels decrease; the mechanistic target of rapamycin (mTOR) and Ras-mitogen-activated protein kinase (MAPK) pathways are inhibited, reducing proliferative signals in neoplastic cells. This situation could potentially decrease the overly active proliferation signal in BC, particularly within the PI3K-Akt-mTOR pathway, which is frequently activated in this type of cancer. In normal cells, mechanisms that safeguard against food scarcity are initiated; DNA repair capabilities are enhanced, oxidative stress is regulated, and cells exhibit heightened resistance to cytotoxic therapies [268].

Its significant impacts on the immune system include a reduction in immunosuppressive cell count, an increase in lymphoid progenitor regeneration, and an enhancement of cytotoxic T-cell activity, hence improving the efficacy of immunotherapy [268,269]. BC, a malignancy linked to persistent inflammation, is significantly influenced by inflammatory pathways, including NF-κB, IL-6, and TNF-α, which are crucial in both tumor initiation and advancement. The fasting-mimicking diet (FMD) potentially mitigates the inflammatory microenvironment within the bladder mucosa, which is maintained by carcinogenic agents, through its capacity to reduce systemic inflammation, inhibit NF-κB signaling, and lower the levels of proinflammatory cytokines.

Furthermore, the immune-modulating properties of the FMD are particularly relevant considering the responsiveness of BC to immunotherapeutic approaches. Cycles of fasting-mimicking diets may augment T-cell functionality, decrease the prevalence of immunosuppressive cells, and foster a more robust T-cell response within the tumor microenvironment. Consequently, this offers a biological rationale that could theoretically amplify the efficacy of immunotherapy, especially in BC patients undergoing treatment with PD-1/PD-L1 inhibitors.

The effects of the FMD extend beyond cancer biology; it also stimulates pathways linked to longevity. Activation of anti-aging systems, including sirtuins, FOXO transcription factors, and AMPK, enhances cellular stress resilience while reducing the buildup of aging-associated mutations and epigenetic decline [270,271,272].

### 4.6. Mediterranean Diet

The Mediterranean diet (MD) is characterized by an abundance of fruits, vegetables, whole grains, legumes, nuts, fish, and seafood, particularly emphasizing the intake of monounsaturated fatty acids from olive oil while restricting the intake of red meat, processed foods, and refined sugars [273]. The MD is receiving heightened attention for its cardiometabolic advantages and potential to reduce cancer risk. The impact of the MD on BC is elucidated mainly through antioxidant, anti-inflammatory, and metabolic regulating pathways [12,274].

Monounsaturated fatty acids and omega-3 PUFAs, sourced from foods like olive oil and fish, safeguard cell membrane integrity, inhibit the synthesis of inflammatory cytokines (IL-6 and TNF-α), and reduce chronic inflammation within the tumor microenvironment. This facilitates a reduction in immunological pressure in the bladder mucosa and an increase in cytotoxic T-cell responses. Therefore, the process of cancer development might slow down, and the signals that encourage the tumor microenvironment to grow could also weaken [275,276].

Fruits, vegetables, and whole grains, which provide the foundation of the diet, are abundant in fiber, vitamins (notably C, E, and folate), and phytochemicals. Polyphenols, flavonoids, and carotenoids mitigate DNA damage by neutralizing free radicals [275]. Oxidative DNA damage plays a crucial role in this cancer type, given the bladder epithelium’s continuous exposure to carcinogens like aromatic amines, nitrosamines, and cigarette smoke metabolites via urine contact [23]. The MD could potentially mitigate oxidative stress-induced DNA breaks within the bladder mucosa by decreasing reactive oxygen species levels. Consequently, its antioxidant properties might offer a significant protective mechanism for the repair of oxidative damage caused by reactive carcinogens present in urine.

The MD’s high fiber content enhances microbiota diversity and increases SCFA synthesis [19]. Butyrate specifically functions as a histone deacetylase inhibitor in epithelial cells, modulating gene expression, facilitating apoptosis, and enhancing cellular differentiation. These pathways, facilitated by the microbiome, enhance immune activities, providing systemic protection against BC. This could represent an additional means of mitigating the initial risk of BC, achieved by transforming the potential carcinogens that are in contact with the bladder epithelium into a less harmful metabolic variant [277]. Furthermore, a meta-analysis by Dianatinasab and associates indicated that the MD offers a protective benefit against BC [12].

The MD possesses a low glycemic load. Restricting refined carbs mitigates fluctuations in insulin and IGF-1, thus curtailing the excessive activation of the PI3K/Akt/mTOR pathway, a crucial regulator of tumor cell proliferation [275,278]. In conclusion, the MD appears to activate various protective mechanisms against BC. These mechanisms provide protection against BC via various pathways, including the expedited detoxification of carcinogens, alteration of the tumor microenvironment, inhibition of proliferative signals through glycemic regulation, and enhancement of the immune response by enriching the microbiota [19,275].

### 4.7. Alternative Diets: Restrictive Models

Restrictive dietary models include strategies that restrict particular macronutrients, micronutrients, or total calorie consumption. The objective is to impede the proliferation and advancement of BC cells by targeting their energy requirements [19,237,279].

Calorie restriction is the most extensively researched restrictive paradigm. Caloric restriction, which seeks to reduce caloric consumption by roughly 10–40%, necessitates a balanced intake of nutrients to avert malnutrition [280]. During periods of caloric restriction, when carbohydrate reserves are low, the body compensates by metabolizing lipids through fatty acid oxidation (FAO) within hepatocytes to generate energy. This process results in the production of substantial quantities of acetyl-CoA and ketone bodies, specifically acetoacetate, acetone, and β-hydroxybutyrate, which can then be utilized as energy sources by various tissues, including the heart, brain, and muscle [226]. Decreasing calorie consumption reduces insulin and IGF-1 levels, thereby inhibiting the PI3K/Akt/mTOR signaling pathway. This route is a primary mechanism facilitating proliferation in BC cells [281,282]. Restricting calories also promotes autophagy and DNA repair pathways through increased AMPK activation. Although normal cells benefit from this adaptability, tumor cells are unable to maintain proliferation due to energy deficiency and consequently undergo apoptosis [281,283]. Moreover, reduced production of inflammatory cytokines undermines immunological pressure within the tumor microenvironment and enhances susceptibility to immunotherapies [281].

The proposed mechanism by which calorie restriction (CR) affects BC centers on metabolic and inflammatory regulation, specifically targeting the core biological factors that drive tumor development. CR potentially diminishes the proliferative advantage of tumor cells by suppressing the PI3K–Akt–mTOR pathway, a pathway often activated in BC, through reductions in insulin and IGF-1 levels. Concurrently, its capacity to decrease oxidative stress and inflammation may impede the accumulation of DNA damage within the bladder epithelium, an area consistently exposed to urinary carcinogens. Furthermore, by promoting apoptosis, CR may decelerate carcinogenesis via early cellular repair processes, while its immune-enhancing properties could bolster the antitumor response by mitigating immune suppression within the tumor microenvironment. Consequently, the cumulative effect of these mechanisms suggests that calorie restriction may offer a protective or tumor-suppressive benefit in BC through metabolic, inflammatory, and immunological pathways.

Carbohydrate restriction directly influences the energy metabolism of neoplastic cells. Numerous tumor cells, such as those in BC, depend on aerobic glycolysis for energy generation, as elucidated by the “Warburg effect” [284]. Glycolysis also produces byproducts like lactate, which can help cancer grow and spread. These byproducts can then be used in the tricarboxylic acid cycle to provide energy for tumors. In BC, cancer cells convert pyruvate into lactate, relying mostly on glycolysis for energy, even when oxygen is present. In this process, glucose enters the cell through the GLUT1 transporter. Then, the LDH-A enzyme converts pyruvate into lactate, instead of using it in mitochondria. Lactate is then released from the cell through MCT4, and nearby cells can take it in through MCT1, using it as an energy source [249,285]. Therefore, a metabolic cooperation develops between the glycolytic and oxidative areas within the tumor. The accumulation of lactate acidifies the tumor microenvironment and degrades the extracellular matrix, thereby promoting the dissemination of malignant cells into adjacent tissues [249]. This process is evident in both superficial and muscle-invasive BCs. A prevalent and critical metabolic weakness in this malignancy is the overexpression of the LDH-A enzyme. Consequently, inhibiting LDH-A impedes the ability of cancer cells to maintain energy production, whereas normal bladder cells retain their capacity for mitochondrial energy generation. Therefore, therapeutic strategies that target glycolysis and lactate metabolism in BC offer promising avenues for treatment.

Limiting carbohydrates decreases glucose availability, resulting in an energy deficit and metabolic stress on neoplastic cells. Throughout this process, ROS levels in mitochondria increase, DNA damage accumulates, and apoptosis is initiated [209,286]. A reduced carbohydrate intake results in decreased levels of insulin and IGF-1. This leads to the inhibition of the PI3K/Akt/mTOR pathway, which facilitates proliferation. This leads to cell cycle arrest and decreased proliferation [284,286].

Protein restriction, particularly the limitation of animal-derived proteins, is a strategy of interest in cancer biology. Some studies suggest that low-protein diets inhibit tumor growth by increasing immune surveillance through downregulation of the IGF/mTOR pathway, reprogramming tumor-associated macrophages, and activating the inositol-requiring transmembrane kinase/endoribonuclease 1α (IRE1α)-dependent unfolded protein response. Other studies, however, have shown that protein-rich diets protect against tumor growth and initiation and may prevent cancer through amino acid-mediated activation of mTOR [40,41,287]. Therefore, tumors likely respond heterogeneously to protein restriction, directing growth signaling and tissue biosynthesis in some cases, but supporting tissue homeostasis to disrupt tumor growth in others [19,41,288]. The potential mechanism of protein restriction in BC is shaped primarily by amino acid signaling, IGF-1 levels, mTOR activity, and carcinogen metabolism. In BC, the PI3K–Akt–mTOR axis is frequently overactive, supporting cell proliferation, metastatic potential, and glycolysis dependence. Protein restriction also reduces IGF-1 levels; since IGF-1 is an important mediator of proliferation and anti-apoptotic signals in BC cells, a decrease in IGF-1 may directly slow tumor growth. Protein restriction metabolically influences nitrogen metabolism. The accumulation of metabolites, including urea and ammonia, generated during protein catabolism might impose further strain on the bladder mucosa.

High-protein diets, especially those abundant in red and processed meats, have been linked to a heightened risk of BC [41,47,287]. The iron and nitrate/nitrite preservatives present in red and processed meat products are converted into N-nitroso compounds when they come into contact with urine, leading to the formation of DNA adducts within the bladder mucosa [41]. Protein restriction might offer a direct protective benefit by reducing exposure to carcinogenic metabolites. Moreover, protein restriction could aid in the restoration of the epigenetic equilibrium [289]. A crucial consideration is that protein restriction might enhance chemotherapy sensitivity by compromising the antioxidant defenses within tumor cells [19]. Metabolically, the restriction of protein intake influences nitrogen metabolism. The accumulation of byproducts like urea and ammonia, which are generated during protein breakdown, can potentially exacerbate stress on the bladder lining. By preventing the buildup of these metabolites, protein restriction helps to mitigate inflammation and cellular damage [290,291]. Nevertheless, the potential drawbacks of protein restriction, including the risk of sarcopenia, compromised immune function, and reduced treatment tolerance, especially in individuals receiving chemotherapy or immunotherapy, must also be taken into account [292,293].

#### 4.7.1. Amino Acid Restriction

Amino acid restriction attempts to limit essential components required for the fast development of cancer cells. Highly proliferative tumor cells utilize amino acids for protein synthesis, nucleotide and lipid biosynthesis, epigenetic regulation, and the preservation of redox homeostasis. Consequently, restricting specific amino acids may inhibit carcinogenesis by reducing the metabolic flexibility of tumor cells [19,23,294].

Methionine restriction is among the most extensively researched targets. Methionine is a crucial amino acid for one-carbon metabolism and DNA methylation [295]. Excessive methionine consumption may increase oncogene expression and the proliferative capacity of tumor cells by elevating DNA and histone methylation [296]. Methionine restriction interrupts this cycle, resulting in DNA hypomethylation and epigenetic reprogramming. This may result in the reactivation of tumor-suppressor genes and the inhibition of oncogenes [296,297]. Moreover, methionine reliance is characterized as a metabolic weakness in numerous tumor cells; whereas normal cells may acclimate to reduced methionine levels, cancer cells are unable to maintain development. Methionine restriction has been documented to inhibit proliferation and halt the cell cycle in the G1 phase in BC cells [296,298].

Methionine is a precursor to S-adenosylmethionine (SAM), which is essential for polyamine synthesis, methylation reactions, and the production of glutathione and cysteine. Changes in diet can affect these processes, leading to the creation of various intermediate metabolites that are crucial for how cells function [227]. Methionine is converted to S-adenosylmethionine (SAM) by methionine adenosyltransferase (MAT). Mammals have three types of MAT isoenzymes: MAT1A, MATII, and MATIII. The expression of MAT1A is linked to slower tumor growth, possibly by promoting growth inhibition, angiogenesis, and apoptosis [19]. Elevated MAT2A expression correlates with heightened cancer cell proliferation, inhibited apoptosis, and accelerated carcinogenesis [227]. BC cells exhibit sensitivity to methionine, a consequence of heightened methionine cycle activity, increased S-adenosylmethionine (SAM) production, and aberrant DNA/RNA methylation. Given the intersection of methionine metabolism with numerous essential biological processes in BC, the modulation of methionine availability or flux may influence bladder tumor biology. Increased methionine levels could potentiate epigenetic silencing in bladder tumor cells through the accumulation of SAM; this process may subsequently elevate methylation and silence tumor-suppressor genes, including p16, RASSF1A, and APC. Conversely, methionine restriction might reduce DNA methylation, thereby alleviating this silencing and facilitating the reactivation of tumor-suppressor genes.

The limitation of serine and glycine is significant in nucleotide biosynthesis [299,300]. Serine participates in one-carbon metabolism through the folate cycle, enabling the production of nucleotides essential for DNA/RNA [299], whereas glycine is essential in glutathione synthesis and the preservation of intracellular redox homeostasis [301]. The limitation of these amino acids inhibits DNA replication in cancer cells, resulting in cell cycle arrest. Moreover, diminished synthesis of reduced glutathione increases vulnerability to oxidative stress, thereby weakening cancer cell defenses. This may enhance the efficacy of chemotherapy and radiotherapy [302]. Reducing serine and glycine levels slows the growth of BC cells. This happens because it disrupts how nucleotides are made, affects the production of glutathione, and interferes with the epigenetic methylation cycle. As a result, oxidative stress increases, making the cells more metabolically vulnerable. This could be a way that the body suppresses tumors in BC.

Arginine restriction represents a viable and feasible strategy in cancer treatment. Arginine is essential for numerous metabolic processes, encompassing protein synthesis; nitric oxide generation; nucleotide synthesis; and the biosynthesis of polyamines, creatine, proline, glutamate, and urea. Furthermore, arginine can enhance protein translation through the activation of mTORC1 [303]. Notwithstanding its crucial roles, recent findings indicate that products derived from arginine, including carbon monoxide and polyamines, alongside arginine-activated signaling pathways, substantially influence cancer progression [19]. Arginine is crucial for the formation of nitric oxide (NO). Reduced concentrations of NO affect cellular signaling, whereas elevated levels correlate with DNA damage [304]. Tumor cells demonstrate arginine dependency due to compromised arginine metabolism. Restriction of arginine can impede tumor cell proliferation and regulate immune cell activity. Nonetheless, because T-cell proliferation in the immune system relies on the availability of arginine, any restrictions must be administered with caution [19,304]. Arginine, a crucial amino acid, significantly impacts various tumor-promoting factors in BC. These include polyamine synthesis, nitric oxide production, epigenetic regulation, metabolic stress, and immune suppression. Consequently, an excess of arginine could potentially foster tumor growth and inflammation. Conversely, limiting arginine availability might initiate a tumor-suppressive process by inducing metabolic vulnerability, curtailing proliferation, and enhancing the immune response.

#### 4.7.2. Fat Restriction

Fat restriction is an approach that primarily focuses on limiting saturated fatty acids. Dietary fat influences the inflammatory process and may enhance carcinogen production. Moreover, the fatty acid composition may enhance the progression of BC by directly influencing metabolic processes [12,23].

Saturated fatty acids activate TLR4 receptors, promote the NF-κB pathway, and enhance the synthesis of proinflammatory cytokines, including IL-6 and TNF-α. This chronic inflammatory condition enhances immune suppression within the tumor microenvironment and promotes tumor advancement [12,305]. Limiting saturated fatty acids reduces inflammatory processes, leading to a more balanced immunological response in the bladder mucosa. Palmitic acid and similar dietary fats have been shown to promote long-term metastasis, a process dependent on CD36. This occurs through changes in gene expression and chromatin structure, which activate Schwann cells within the tumor. Moreover, increased lipid absorption via CD36 has been linked to CD8+ T-cell dysfunction in various models. As a result, this creates an immunosuppressive tumor microenvironment, which then promotes the initiation of metastasis [226]. Moreover, the composition of dietary lipids influences hormone metabolism. Excessive fat consumption correlates with obesity, hyperinsulinemia, and increased IGF-1 levels. Increased IGF-1 levels activate the PI3K/Akt/mTOR pathways, consequently enhancing cell proliferation [12,23]. Limiting fat intake may inhibit tumor cell proliferation by preventing the excessive activation of these pathways [305].

**Figure 3:**
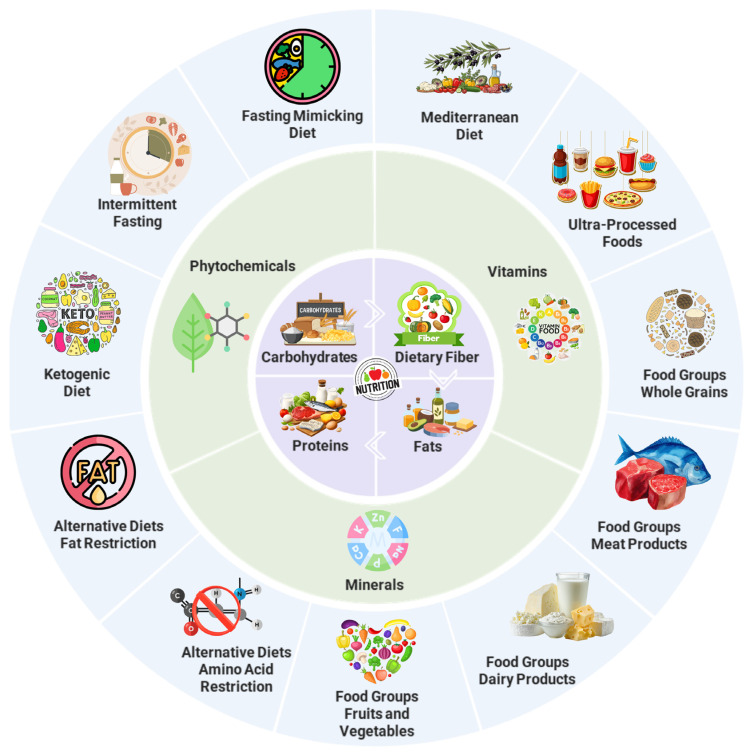
Nutrition-related pathways in the management of BC.

High-fat diets metabolically modify the lipid composition of cell membranes, limiting membrane fluidity, disrupting cellular signaling pathways, and promoting tumor cell proliferation [306,307]. Limiting fat intake may relieve these biophysical alterations. A high-fat diet elevates free fatty acid oxidation, thereby increasing mitochondrial ROS generation. This elevates the probability of DNA damage and mutagenesis [306,307]. Taking all these aspects into account, fat restriction may aid in maintaining cellular integrity by reducing oxidative stress [12]. Fat restriction could potentially impede the initiation and advancement of BC through several mechanisms: mitigating inflammation, decreasing reactive lipid derivatives, disrupting the IGF-1/mTOR pathway, reducing the burden of carcinogen metabolites, and inhibiting lipid-derived proliferative signals. The nutrition-related pathways in the management of BC are presented in Figure 3.

A comprehensive understanding of how dietary factors influence BC risk, clinical outcomes, and treatment effectiveness is essential for the formulation of effective preventive and therapeutic interventions. Research findings indicate that specific dietary components and patterns may either promote the development of BC or exert protective effects. Diets characterized by low fruit and vegetable consumption are linked to an elevated risk, and high-fat diets are also associated with an increased risk. Furthermore, certain nutraceuticals and supplements, which contain bioactive compounds, can potentially influence BC biology, thereby affecting both tumor growth and the response to treatments.

## 5. Precision Nutrition, Potential Challenges, and Future Perspectives

The adaptation of precision nutrition recommendations and interventions based on individual genetic, epigenetic, metabolomic, microbiome, and lifestyle profiles has gained momentum with advancements in research, technology, and services in these areas. However, while technological and scientific advancements promise unprecedented personalization, significant challenges remain. This section critically examines these challenges and explores emerging future directions [308]. Effective precision nutrition recommendations necessitate the integration and harmonization of multi-omics data, encompassing genomes, epigenomics, metabolomics, proteomics, and the microbiome. The diversity of data formats, varying dimensions (ranging from gene variations to metabolites), and statistical intricacies may result in challenges in integration [309]. The lack of standard protocols for data collection, explanation, and sharing in precision nutrition studies weakens reproducibility and comparability between studies and undermines standardization (biological samples (e.g., stool, blood, urine), circadian rhythms, nutrient intake, stress, etc.) [137]. Studies and recommendations based on tailored and precision nutrition may not show sufficient impact on public health if they remain individual-based and avoid a community-based approach. In this case, it is thought that sectors with economic, social, and cultural power could exploit tailored and precision nutrition studies [310]. Findings on tailored and precision nutrition should be standardized to account for variability among individuals, considering the different responses that they exhibit due to environmental exposures, epigenetic modifications, or variations in the gut microbiota [200].

Although tailored and precision nutrition tackles issues pertaining to enhanced health equity, it frequently emphasizes individual-level determinants that influence health [311]. The majority of tailored and precision nutrition cohorts are based in Europe, and it is believed that the underrepresentation of various backgrounds may increase inequities in nutritional advising and health outcomes [312]. Nevertheless, tailored and precision nutrition frequently depends on expensive technologies and specialized analyses (e.g., omics profiling and continuous glucose monitoring), which may pose obstacles for communities and people lacking sufficient economic and healthcare resources. If not managed meticulously, these techniques may exacerbate health disparities [313].

Concerns arise regarding the ethics, legal aspects, privacy, data security, informed consent, and potential psychological effects of using genetic, epigenetic, genomic, metabolomic, and metagenomic information, as well as other highly sensitive personal data (a biopsychosocial model encompassing psychological and sociological factors) [153,314]. In studies based on tailored and precision nutrition, participants must provide informed consent for the use of highly sensitive personal information, and researchers must guarantee ethical, legal, privacy, and data security [315]. However, such studies should prioritize improving public health; present the data obtained transparently and impartially with minimal usage; and continue to strengthen ethics, privacy, and data security to facilitate the integration of tailored and precision nutrition approaches [316]. Furthermore, in studies focused on tailored and precision nutrition, the efficient collection, standardization, integration, security, and analysis of intricate and interrelated data from populations and individuals concerning health, biological, social, behavioral, environmental, and consumer purchasing factors are crucial for supporting evidence-based health outcomes [317]. A further problem in studies focused on tailored and precision nutrition is the accessibility and sharing of data, as the commercial sector and several organizations often refrain from making their data publicly available, utilizing it as a substantial competitive advantage [318]. Data collection by healthcare organizations and professionals, including healthcare practitioners and dietitians/nutritionists, may be associated with a lower perceived risk and higher credibility than that by commercial businesses [319].

The rapid advancement of artificial intelligence (AI) and machine learning (ML) has significantly increased the speed, scope, and depth at which data can be analyzed, integrated, and evaluated. Developments in artificial intelligence and machine learning, particularly in deep learning, which can integrate multimodal datasets, hold promise for more accurate and precise nutritional recommendations [320]. Ensuring sustained user involvement in research utilizing multi-omics data is a crucial yet formidable task; the novelty impact may diminish with time, leading to a decline in user application utilization. Moreover, transforming digital feedback into enduring dietary behavior modification remains a formidable challenge [321]. These systems utilize extensive datasets and machine learning algorithms to fulfill a critical requirement for adaptability by integrating wearable technologies and systems that improve the precision of nutritional intake evaluation with real-time feedback, as well as accounting for variables such as genetics, microbiome composition, and cultural dietary preferences. This facilitates the optimization of nutritional programs among numerous populations [322]. Nonetheless, precision nutrition methodologies, coupled with machine learning, have inaugurated a novel epoch in tailored and precision nutrition interventions by delivering extensive genotypic and phenotypic big data regarding individual dietary interactions [323]. The data-driven solutions offered facilitate accurate nutritional recommendations aimed at enhancing health outcomes. Moreover, these systems are broadening the scope of nutritional research by tackling pressing global challenges such as the management of malnutrition and diet-related illnesses [324]. The application of big data and machine learning holds significant potential for the advancement of nutritional epidemiology, enhancing the accuracy and validity of tailored and precision dietary assessments (such as food consumption records or frequencies) [325]. Looking ahead, understanding the potential of AI in precision nutrition will be significantly enhanced, providing innovative directions for future research [326]. However, there is a lack of consistent data structuring across scientific fields, and there can be vast differences in the complexity, comprehensiveness, reliability, validity, and transparency of different AI and ML algorithms, models, and tools. It should be noted that cross-functional expertise may be required to develop recommendations; correct algorithms, models, and tools for PN using AI and OR; select and organize data sources; and conduct appropriate analyses [327]. Another critical factor in the effectiveness of tailored and precision nutrition studies is incorporating behavioral science to support effective communication with participants. The suggestions should easily fit into participants’ daily activities and be presented at the right time. The dataset used should be applicable to the individual and population being studied. This may require the information to be transmitted through a technological device. These requirements are important considering the integration of technology and the use of artificial intelligence systems, as there is potential for misinformation [318,328]. Digital tools for precise meal planning, dietary monitoring, and precision nutrition offer enhanced health benefits. The integration of artificial intelligence algorithms and technologies designed to streamline the collection and management of nutritional data from digital apps markedly improves the efficacy of tailored and precision nutrition. Nonetheless, the constraints of these digital applications include data integrity, accessibility, and price, necessitating additional research and development [329].

Clinical trials often do not adequately represent vulnerable or diverse populations (e.g., older adults, low-income groups, and ethnic minorities), raising concerns about the applicability of the findings [330]. Extensive longitudinal cohorts and flexible tailored and precision nutrition frameworks for randomized controlled trials will be essential for establishing sustainable efficacy and feasibility across diverse populations [317]. In studies focused on tailored and precision nutrition, significant limitations are the challenge of accurately assessing people’ eating behaviors, their noncompliance with tailored dietary recommendations, and the insufficiency of precision nutrition guidance in resolving individual-specific issues [331]. Recent technological advancements have led to the development of various devices, including wearables and sensors, which, alongside mobile applications and web-based tools, enable more accurate and objective assessment of dietary intake and eating behavior than traditional self-reporting methods. To address this scenario, it is essential to enhance individuals’ health and technical literacy, as well as to elevate knowledge of tailored and precision nutrition [332]. Challenges and future perspectives in precision nutrition are presented in Table 2.

Tailored and precision nutrition guidance has a bright future for improving health and reducing chronic diseases [336]. To realize this potential, substantial difficulties including technical complexity, insufficient evidence, privacy and equality issues, and integration constraints must be resolved [334]. Converging advancements in multi-omics, artificial intelligence, systems biology, ethical frameworks, and behavioral sciences are facilitating the emergence of tailored and precision nutrition as a scalable, inclusive, and effective platform for health enhancement, rather than a mere exclusive innovation [335].

Clinical applicability, cost-effectiveness, and scalability are critical factors for PN. To ensure clinical usefulness, the functional feasibility of PN should be improved within a structured phased framework that includes extensive multi-omics profiling [337,338]. In BC treatment, PN is of paramount importance as a “first-line” approach that can be implemented without requiring costly infrastructure, is scalable, and utilizes routinely available clinical and dietary data (diet quality, weight monitoring/body composition, comorbidities and treatment context, symptom burden, and standard laboratory parameters when available) [4]. Precision nutrition, in its “second stage,” enables selectively targeting biomarkers or digital phenotyping for higher-risk or complex patients, while in its “third stage,” it gains applicability by integrating multiple omics, microbiome, and individual data obtained through evidence, standardization, and feedback pathways into artificial intelligence decision support [339,340]. This incremental strategy tackles critical implementation obstacles, including the substantial expense of omics profiling, workflow interruptions, and disparities, while preserving the objective of administering appropriate nutritional interventions to patients at the optimal time [138]. Current extensive initiatives focused on forecasting individual reactions to foods and dietary patterns highlight that clinical scalability will require verified algorithms, interoperable data pipelines, and thorough evaluation across varied populations, rather than solely the accumulation of additional molecular data [340,341].

### Practical Implementation Challenges of Precision Dietary Modalities in Bladder Cancer Care

The clinical use of precision nutrition in BC transcends molecular profiling and data integration, necessitating a practical implementation framework that guarantees safety, feasibility, and ongoing adherence [4,342]. The same dietary approach may be supportive in one patient and harmful in another, depending on factors such as nutritional status, comorbidity, metabolic function, and cellular capacity [23]. This precise “patient-specific” modification is a fundamental challenge in precision nutrition and must be expressly addressed to prevent overgeneralization and improve therapeutic applicability.

While popular dietary models such as ketogenic, intermittent fasting, and fasting-mimicking diets highlight the mechanistic rationale behind them, their clinical application in BC requires careful evaluation in terms of feasibility, safety, and individual adaptability; this represents the practical challenges inherent in precision nutrition practice [343]. It is important not only to determine “which dietary model makes metabolic sense in BC”, but also for whom, when, under what monitoring conditions, and with what safety precautions these methods can be applied [344]. In particular, BC disproportionately affects older adults, and many patients present with comorbidities such as metabolic syndrome, diabetes, frailty, risk of sarcopenia, and renal sensitivity; these factors can render a dietary approach clinically unsafe rather than supportive if not individualized [345,346].

Ketogenic diets demonstrate the need to balance molecular potential with clinical limitations. Ketogenic interventions may reduce insulin/IGF-1 signaling and affect inflammatory pathways; however, the literature acknowledges a correlation between ketogenic dietary patterns and an elevated risk of nephrolithiasis, with documented instances of kidney stone complications and associated renal events among ketogenic diet adherents [347]. This is especially pertinent to BC populations, where baseline renal function may be impaired due to age, hydronephrosis, risk of urinary obstruction, recurrent infections, or systemic treatments [348]. Moreover, ketogenic diets may prove challenging to maintain in patients experiencing reduced appetite, treatment-induced nausea, or catabolic stress, necessitating proactive measures to avert inadvertent weight loss and muscle degradation, particularly in weak or sarcopenic individuals [349]. Therefore, ketogenic approaches should be seen as high-intensity precision therapies necessitating tailored selection (considering renal function, history of stones, and nutritional condition), systematic monitoring, and feasibility-conscious design instead of generalized population recommendations.

Intermittent fasting (IF) and related fasting practices are important but require caution. While IF can improve insulin sensitivity and metabolic flexibility, fasting-based strategies in oncology require a careful risk–benefit assessment because repeated calorie restriction can worsen malnutrition and sarcopenia, which are closely associated with poorer tolerance to and worse outcomes from cancer treatment [350,351]. This concern is of paramount importance for BC patients undergoing chemotherapy, immunotherapy, or pre-surgical care, where nutritional reserves and protein adequacy are critically important, and the feasibility of intermittent fasting (IF) should be thoroughly evaluated within the scope of precision medicine and nutrition [352,353].

In preclinical studies, fasting was found to potentiate the effects of several anticancer treatments, and early clinical studies indicated that patients may benefit from modified fasting regimes. A study reported that periodic FMD cycles are feasible and can be safely combined with standard antineoplastic therapies in cancer patients with low nutritional risk [354]. Another study demonstrated that the FMD program was well-tolerated by breast cancer patients, with few or no fasting-related side effects and acceptable weight loss, proving safe. Additionally, it has been reported to potentially enhance the effectiveness of anticancer treatments by reducing IGF1, which is indirectly linked to insulin resistance and inflammation [355]. Nonetheless, significant feasibility restrictions persist for the FMD: obstacles to adherence; inter-individual metabolic variability; and the need for alignment with therapeutic regimens, symptom burden, and patient preferences [356]. These realities highlight the essential role of precision nutrition in dietary approaches.

## 6. Conclusions and Future Directions

In conclusion, precision nutrition for bladder cancer (BC) holds promise in addressing various unanswered questions and delivering health benefits at the community level. Advances in high-performance technologies have created opportunities to explore the roles of various omics data in understanding the pathophysiology and management of complex diseases. This rich and precise data, combined with access to electronic health records and the rapidly increasing power of computational analysis and bioinformatics, forms the foundation for establishing precision nutritional requirements for BC.

This can be achieved by establishing methods for the optimal and accurate determination of food intake and the assessment of nutritional status, and by integrating artificial intelligence and reliable, sustainable wearable and mobile devices.

Specifically, the results of prospective cohort studies and meta-analyses will enable the development of intervention strategies for tailored and precision nutrition, utilizing both traditional and newly developed approaches to obtain robust evidence on the relationship between nutrition and BC.

Additionally, another requirement for BC interventions based on tailored and precision nutrition should be the consideration of differences in ethnic origin and population variables. Different metabolic phenotypes resulting from ethnic origins can respond differently to the consumption of the same nutrients, leading to the release of distinct biomarkers. Future nutritional research should also consider the diverse eating habits and dietary patterns of populations with different ethnic backgrounds.

The future of precision nutrition in BC treatment will emphasize food as medicine/food for optimal health, with solutions that combine shopping lists and meal plans; retailers will play a greater role in helping consumers improve their health (e.g., providing guidance on food choices in store); interest in sustainability will increase consumer demand for plant-based, consciously produced food options; and new regulations will be implemented to keep user data secure and promote appropriate controls for providing medical advice.

The available literature shows that, in individuals with BC, PN can help promote motivation, behavior change, and improved health outcomes; however, more research is needed to demonstrate that PN is more effective than public health recommendations. This type of precision guidance for individuals with BC should be accessible to everyone without being excessively expensive. The successful implementation of such an inclusive approach could significantly improve the nutritional status of a significant number of individuals with BC and potentially have a significant public health impact.

## Figures and Tables

**Table 1:** Dietary bioactive substances influencing cancer-associated miRNAs and pathways with potential significance for precision nutrition in bladder cancer.

	Modulated miRNAs	Level of Evidence (miRNA Modulation)	Principal Pathways	Evidence in Bladder/ Urologic Cancers	Precision Nutrition for Bladder Cancer
Curcumin [169,170,171,172,173,174]	↓miR-21, ↓miR-17-92 cluster (miR-17, miR-19b, miR-92a), ↑miR-34a/c, ↑miR-143, ↑miR-22	Preclinical evidence relevant to urology and bladder; no conclusive clinical validation for miRNA endpoints in BC.	PI3K/Akt/mTOR pathway, apoptosis (Bcl-2, Bax), cell cycle arrest, invasion/metastasis, inflammation, chemoresistance	miR-21 is overexpressed in urologic malignancies, particularly BC, and promotes proliferation, invasion, and resistance to therapy. Curcumin inhibits miR-21 and reinstates PTEN/Akt signaling in many tumor models, and is suggested as a supplementary agent in BC treatment.	In individuals exhibiting molecular profiles marked by elevated miR-21 and active PI3K-Akt signaling, dietary regimens rich in curcumin may augment sensitivity to intravesical or systemic chemotherapy and facilitate chemoprevention.
Resveratrol [175,176,177,178]	↓miR-21, ↑miR-34a, ↑miR-663, modulation of miR-96 and miR-146a	Preclinical (BC cell-line evidence for miR-21 →Akt/Bcl-2 axis; clinical translation unproven).	Akt/Bcl-2 axis, apoptosis, cell cycle arrest, angiogenesis (HIF-1α/VEGF), invasion/metastasis	Resveratrol induces apoptosis and reduces proliferation in BC cell lines through miR-21-mediated control of Akt/Bcl-2, while also disrupting stromal-tumor interactions and hypoxia-driven angiogenesis.	In BC patients exhibiting anti-apoptotic miRNA signatures (e.g., elevated miR-21, Bcl-2-mediated), resveratrol-rich meals or supplements may be regarded as adjuncts to standard treatment to enhance apoptosis and reduce angiogenesis, within safety parameters.
Quercetin [179,180,181,182]	↓miR-27a, ↓miR-21, modulation of miR-19b, miR-26b, miR-203, miR-96, miR-143, miR-133b	Preclinical (mostly indirect/heterogeneous across cancers; limited direct bladder-cancer miRNA validation).	EMT and invasion (MMPs, MT1-MMP), cell cycle regulation, oxidative stress response, apoptosis, chemoprevention	Quercetin inhibits oncogenic miRNAs (e.g., miR-27a) and enhances tumor-suppressor miRNAs in many malignancies. Experimental results in BC and other urologic malignancies indicate that quercetin-based combinations suppress proliferation, invasion, and autophagy via miRNA-dependent pathways.	For individuals exhibiting miRNA profiles that suggest increased EMT and invasion (e.g., elevated miR-27a, diminished miR-143/133b), a greater consumption of quercetin-rich foods (such as onions, apples, and dark berries) may be used as an adjunct to a precision nutrition strategy designed to mitigate invasiveness.
ω-3 polyunsaturated fatty acids [183,184,185,186]	↓miR-21, modulation of miR-19b, miR-26b, miR-203, miR-92a, miR-17-92 and miR-106b in combination with fermentable fiber	Preclinical (miR-21/PTEN mechanistic evidence largely outside BC; BC evidence mainly animal/chemoprevention and pathway-level)	Cell membrane remodeling, apoptosis (PTEN/Bcl-2), inflammation, stem-cell and chemoresistance pathways	ω-3 PUFAs reduce oncogenic miR-21 and increase PTEN expression in breast and other cancers, lowering proliferation and potentially enhancing treatment response. Diets combining ω-3 fatty acids with fermentable fibers modulate a panel of stem cell and tumor-suppressor miRNAs (miR-19b, miR-26b, miR-203, etc.).	For BC patients with systemic inflammation and miR-21-driven resistance pathways, ω-3-enriched dietary patterns (e.g., fatty fish, fish oil) may be tailored to down-regulate oncogenic miRNAs and improve the efficacy of systemic therapies.
Dietary fiber SCFAs (butyrate, etc.) [187,188,189,190]	↑miR-203, ↑miR-22, ↑miR-19b/26b (with ω-3), ↓miR-92a	Preclinical (primarily mechanistic evidence from GI/cancer models; BC-specific miRNA evidence limited/indirect).	Inhibition of histone deacetylase, apoptosis (miR-203), immunological regulation, Wnt/β-catenin pathway, inflammation, chemoresistance	The fermentation of fiber into butyrate enhances the expression of tumor-suppressor miRNAs (miR-203, miR-22) and decreases the expression of the oncogenic miR-92a, resulting in decreased proliferation, invasion, and increased apoptosis in cancer models. Fiber and fish oil diets synergistically influence a miRNA panel with chemoprotective properties.	In BC survival, a high-fiber, plant-based diet may indirectly influence systemic and gut-derived miRNA profiles (via SCFAs), aiding in inflammatory regulation and metabolic health, and potentially enhancing responses to immunotherapies and systemic treatments.

Footnote for Table 1: The level of evidence denotes the most robust evidence substantiating the documented diet–miRNA correlations and subsequent pathways. For the majority of bioactive drugs, miRNA regulation is primarily substantiated by preclinical models (in vitro and/or animal research); data from human trials is scarce and frequently indirect. Consequently, clinical translation in bladder cancer remains investigational and should not be construed as demonstrating therapeutic efficacy. ↑, increased miR expression; ↓, decreased miR expression; BC, bladder cancer; miRNA or miR, microRNA; SCFAs, short-chain fatty acids; PTEN, Phosphatase and Tensin Homolog; PUFAs, polyunsaturated fatty acids; Bcl-2, Beclin 2; EMT, epithelial–mesenchymal transition; MMP, matrix metalloproteinase; MT1-MMP, membrane type 1 matrix metalloproteinase; Akt, enzymes help to transmit signals inside cells; HIF-1, hypoxia-inducible factor 1; VEGF, vascular endothelial growth factor; BAX, BCL2 Associated X, Apoptosis Regulator.

**Table 2:** Challenges and future perspectives in precision nutrition for bladder cancer.

Variables	Challenges	Future Perspectives	References
Tailored and precision nutrition dietary recommendations for BC	Heterogeneity in metabolic outputs; limited generalizability of prediction models; high cost of omics profiling; risk of inequality	AI-driven multi-modular models; economical biomarkers; incorporation of varied cohorts	[318,328]
Use of digital health technologies in precision nutrition for BC	Inadequate food intake reporting; fluctuating accuracy of wearable devices; inadequate user compliance; unsustainable data ecosystems	Passive automatic monitoring (food image recognition, CGMs); precise feedback	[322,331,332]
Limitations of clinical research on precision nutrition for BC	Small sample sizes; short trial durations; heterogeneous populations; ethical/privacy concerns	Extensive longitudinal randomized controlled trials (e.g., NIH Nutrition for Precision Health); standardized data metrics; comprehensive data management	[317,330,333]
Future research trends in precision nutrition for BC	Significant challenges such as lack of evidence, privacy and equality concerns, and integration limitations	Integrating advancements in multi-omics, artificial intelligence, systems biology, ethical frameworks, and behavioral sciences facilitates the emergence of tailored and precision nutrition as a scalable, inclusive, and successful health promotion platform	[334,335]

BC, bladder cancer.

## Data Availability

Our review did not examine or produce any publicly accessible archived datasets or links.

## Abbreviations

The following abbreviations are used in this manuscript:

BC	Bladder Cancer
PN	Precision Nutrition
MIBC	Muscle-Invasive Bladder Cancer
NMIBC	Non-Muscle-Invasive Bladder Cancer
GI	Glycemic Index
SCFAs	Short-Chain Fatty Acids
MUFAs	Monounsaturated Fatty Acids
PUFA	Polyunsaturated Fatty Acid
SFA	Saturated Fatty Acid
TFA	Trans Fatty Acid
IGF-1	Insulin-Like Growth Factor 1
ROS	Reactive Oxygen Species
EMT	Epithelial–Mesenchymal Transition
NF-κB	Nuclear Factor-Kappa B
IL-6	Interleukin-6
TNF-α	Tumor Necrosis Factor-Alpha
PD-1	Programmed Cell Death
IF	Intermittent Fasting
FMD	Fasting-Mimicking Diet
MAPK	Mitogen-Activated Protein Kinase
DNA	Deoxyribonucleic Acid
RNA	Ribonucleic Acid
GSTM1	Glutathione S-Transferase Mu 1
HIF-1	Hypoxia-Inducible Factor 1
VEGF	Vascular Endothelial Growth Factor
MD	Mediterranean Diet
MAT	Methionine Adenosyltransferase
AI	Artificial Intelligence
